# Natural variation in *CTF1* conferring cold tolerance at the flowering stage in rice

**DOI:** 10.1111/pbi.14600

**Published:** 2025-01-29

**Authors:** Jingfang Dong, Shaohong Zhang, Haifei Hu, Jian Wang, Risheng Li, Jing Wu, Jiansong Chen, Lian Zhou, Yamei Ma, Wenhui Li, Shuai Nie, Shaokui Wang, Guiquan Zhang, Bin Liu, Junliang Zhao, Tifeng Yang

**Affiliations:** ^1^ Rice Research Institute Key Laboratory of Genetics and Breeding of High Quality Rice in Southern China (Co‐construction by Ministry and Province), Ministry of Agriculture and Rural Affairs, Guangdong Key Laboratory of New Technology in Rice Breeding, Guangdong Rice Engineering Laboratory, Guangdong Academy of Agricultural Sciences Guangzhou China; ^2^ South China Agricultural University Guangzhou China

**Keywords:** cold tolerance, QTL, single segment substitution line, haplotype analysis, functional site, rice

## Abstract

Improving cold tolerance at the flowering stage (CTF) in rice is crucial for minimising yield loss, making the identification and application of cold‐tolerant genes and QTLs imperative for effective molecular breeding. The long lead time, dependence on cold treatment conditions, and the inherent complexity of the trait make studying the genetic basis of CTF in rice challenging. To date, the fine‐mapping or cloning of QTLs specific to CTF has not yet been achieved. In this study, single segment substitution lines (SSSLs) were constructed using HJX74 as the recipient and IR58025B, known for good CTF, as the donor. This approach led to the identification of two cold tolerance QTLs, *qCTF3* and *qCTF6*, in rice. *qCTF6* has promising breeding potential. Further, we identified the causal gene *CTF1* underlying *qCTF6* through map‐based cloning*. CTF1* which encodes a conserved putative protein, has two SNPs within its coding sequence that influence CTF in rice. Additionally, genetic variations in the promoter of *CTF1* also contributes to CTF. Thirteen variant sites of *CTF1* in the four cold tolerance SSSLs are consistent with the IR58025B. Moreover, we analysed 307 accessions to characterise haplotypes based on the 13 variation sites, identifying five distinct haplotypes. The selection and evolutionary analysis indicate that the cold‐tolerant haplotype of *CTF1* is a newly generated mutation that has undergone selection in *japonica* during domestication. This study not only provides a novel favourable gene for molecular breeding of CTF but also highlights the potential of *CTF1* in advancing rice breeding.

## Introduction

Rice, a staple food for over half of the world's population, originates from tropical or subtropical areas and is sensitive to low temperatures (Ji *et al*., 2021; Peng *et al*., 2024). Cold injury is a long‐term threat to the issue of rice security in China and many other countries in the world, such as Japan, North Korea, Nepal, Madagascar, Bengal, Pakistan, Indonesia, Peru, etc. (Kaneda and Beachell, 1974). China alone experiences an annual rice yield loss of 3–5 million tons due to low temperatures (Liu and Deng, 2009; Zhang *et al*., 2017).

Rice cultivation in China spans a wide latitudinal range, from 53°27′ to 18°90′ north, with regions including North China, Northeast China, Northwest China, the middle and lower reaches of the Yangtze River, Southwest China, and Southern China. The extensive planting environment makes it possible for rice to encounter cold injury at different growth stages (Zhang *et al*., 2017). Among them, cold injury at the flowering stage, characterised by delayed heading, disrupted flowering cycles, sporadic flowering throughout the day, poor pollen dispersal, and arrested development of fertilised zygotes, seriously threatens the yield of rice. Guizhou Province experienced a 21% reduction in production due to cold injury during the flowering period in 2002 (Liu and Deng, 2009). In Southern China's double‐season rice regions, the late‐season rice often suffers from cold injury caused by ‘Cold Dew Winds’ at the flowering stage that led to severe yield losses. Understanding the genetic basis of cold tolerance at the flowering stage (CTF), identifying QTLs/functional genes, and conducting molecular breeding are efficient and effective strategies for improving rice CTF. However, no functional genes for CTF in rice have been reported until now.

The determination of CTF in rice is challenging due to its dependence on the flowering stage and the long growth cycle, necessitating extended research periods. Additionally, CTF assessment requires specific low‐temperature conditions, which can only be reliably provided by large‐scale artificial climate chambers. In addition, CTF is a complex trait influenced by genotype, environmental factors, and their interactions, which complicates and slows the elucidation of its genetic basis. For these reasons, reports on CTF genes in rice remain limited.

Cold tolerance at the flowering stage is distinct from cold tolerance at the booting stage (CTB), which mainly affects pollen fertility and thus yield differences (Zhang *et al*., 2017). Significant advancements have been made in understanding the genetic underpinnings of CTB in rice. Initially, Takeuchi *et al*. (2001) and Saito *et al*. (2001) identified QTLs for CTB on chromosomes 1, 4, 7, and 11, respectively. Advances in molecular marker technology and the use of diverse bi‐parental mapping populations have led to the identification of numerous QTLs for CTB across all 12 rice chromosomes (Andaya and Mackill, 2003; Yang *et al*., 2015; Zhang *et al*., 2014), and only seven have been fine mapped, including *Ctb1* (Saito *et al*., 2004), *qCTB8* (Kuroki *et al*., 2007), *qCTB7* (Zhou *et al*., 2010), *qCT‐3‐2* (Zhu *et al*., 2015), *qLTB3* (Ulziibat *et al*., 2016), *qCTB10‐2* (Li *et al*., 2018) and *qCTB1t* (Guo *et al*., 2020). Aside from using bi‐parental mapping populations, genome‐wide association study (GWAS) is also an effective approach for cold tolerance QTL mapping in rice (Cui *et al*., 2013; Guo *et al*., 2020; Lou *et al*., 2022; Xiao *et al*., 2018). Although numerous QTLs for CTB have been mapped, the cloning of these loci has been limited. Notably, the F‐box protein gene has been identified as the causal gene for the QTL *Ctb1* (Saito *et al*., 2010). Additionally, the gene *CTB4a*, which encodes a conserved leucine‐rich repeat receptor‐like protein kinase, has been successfully cloned from the QTL *qCTB4‐1* (Zhang *et al*., 2017). Further, the sterol glycosyltransferase gene, *CTB2*, has been identified through a joint approach combining linkage analysis and GWAS, and intriguingly, it is also located within the *qCTB4‐1* (Li *et al*., 2021b). Another gene, *LOC_Os07g07690*, which encodes a protein containing a PHD‐finger domain, has been pinpointed as the causal gene for *qCTB7* (Yang *et al*., 2023a). Similarly, *COG3*, encoding a putative calmodulin‐binding protein, has been identified as the causal gene for *qCTBS11* (Liu *et al*., 2024). The study of the *ltt1* mutant has shed light on the role of a 5561‐bp genomic DNA segment in conferring CTB (Xu *et al*., 2020). Furthermore, genes such as *OsAPX1* (Sato *et al*., 2011), *bZIP73* (Liu *et al*., 2019), *WRKY53* (Tang *et al*., 2022), *OsMAPK3* (Lou *et al*., 2022), *OsLEA9* ^*KL*^ (Lou *et al*., 2022) have been demonstrated to play a role in CTB through the evaluation of corresponding transgenic rice plants. However, despite these advancements, there remains a dearth of solid genetic evidence that elucidates the functional sites of these genes.

Based on the confirmation of causal genes, further identification of their functional site will help to accurately select favourable alleles among breeding lines with rich genetic backgrounds, thus improving the efficiency of molecular breeding. Understanding the origin of cold tolerance genes is crucial, as both ancestral standing variation and newly generated mutations contribute to rice's adaptation to different ecological zones. Clarifying the origin and selective utilisation of target genes can enhance our understanding of their role in rice evolutionary and accelerating their application potential.

In this study, in order to explore genes for CTF with breeding applications value in rice, SSSLs derived from *indica* IR58025B as the donor and *indica* HJX74 as the recipient were used for cold tolerance evaluation. Two QTLs, *qCTF3* and *qCTF6*, were mapped using SSSL; furthermore, the causal gene, *CTF1*, underlying *qCTF6* was functionally confirmed. *CTF1* regulates the CTF through both its promoter and coding regions. Phylogenetic and geographic distribution analyses suggested that *CTF1* is a newly generated mutation retained by selection during *japonica* domestication. These findings reveal the function of *CTF1* in cold tolerance and its potential for molecular design in rice breeding.

## Results

### The mapping of QTL

In order to find stable QTL for CTF in rice, the 21 SSSLs constructed with HJX74 as the recipient and IR58025B (cold tolerance) as the donor (Yang *et al*., 2017; Zhang, 2021; Zhang *et al*., 2004) were used for the mapping QTL associated with CTF. The CSSL W12‐25 had a significantly higher seed setting rate after cold treatment than that of HJX74 in three independent experiments (*P* < 0.01) (Figure S3a,b). Based on the genome assembly sequence comparison between W12‐25 (this study) and HJX74 (Li *et al*., 2021a), W12‐25 carried six chromosomal segments from IR58025B, which were distributed on chromosomes 1, 3, 6, 10 and 12 (Figure S3c).

**Figure 1:**
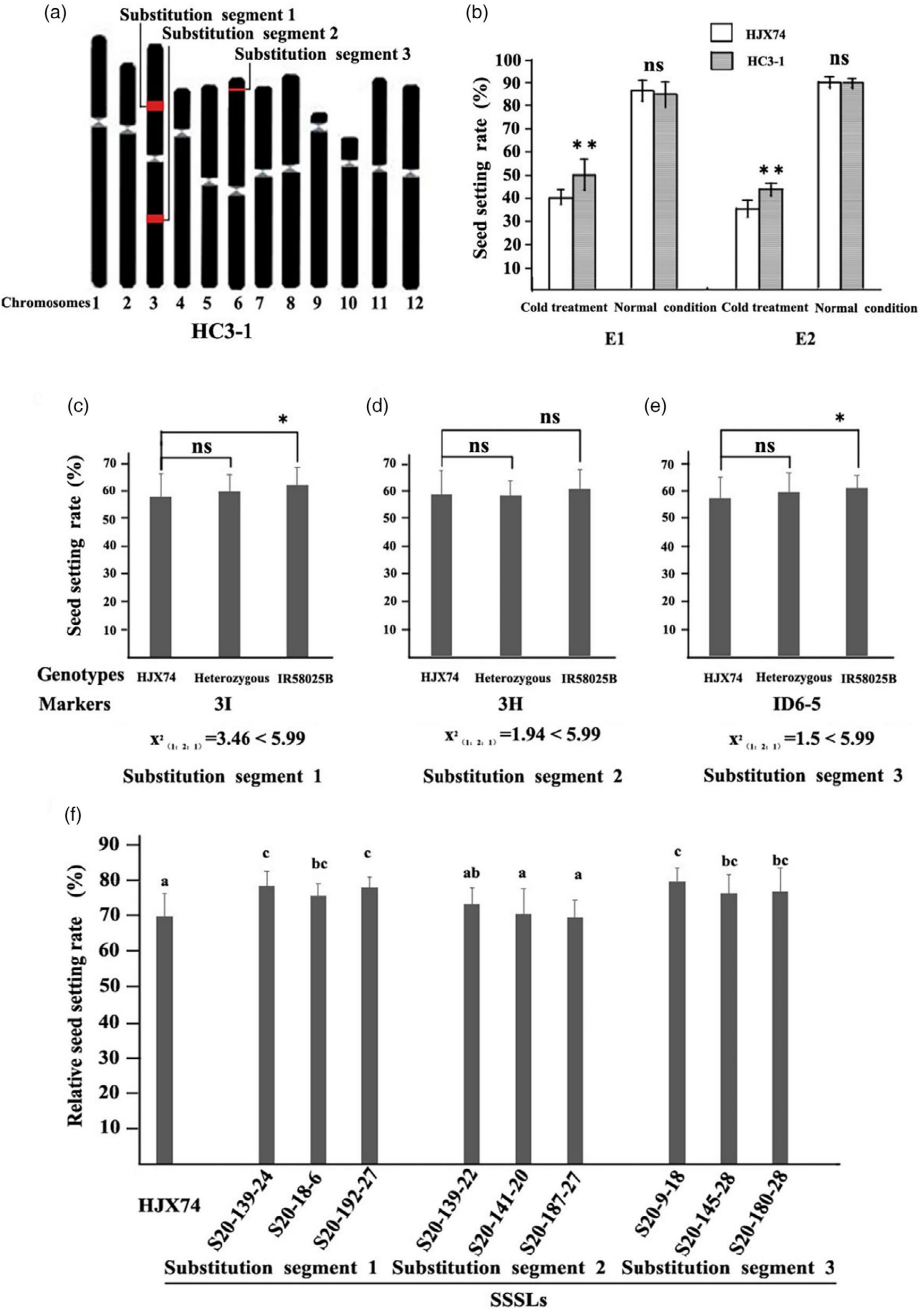
*qCTF3* and *qCTF6* conferring cold tolerance at the flowering stage. (a) The substitution segments of HC3‐1. Red and black blocks on chromosomes represent genotypes of IR58025B and HJX74, respectively; (b) the seed setting rate of HC3‐1 and HJX74 in the two independent experiments; differences in seed setting rate after cold treatment among the three types genotype of substitution segment 1 (c), substitution segment 2 (d), substitution segment 3 (e) in the F_2_ population of HC3‐1/HJX74. * and ** indicate significance of difference at *P* < 0.05 and *P* < 0.01, respectively, based on t‐test; ns means no significant difference; (f) the relative seed setting rate of SSSLs and HJX74 after cold treatment. Different alphabet letters denote differences at 0.05 level of significance in Duncan test. The values of relative seed setting rate are presented using mean and standard deviations.

To narrow down the range of cold tolerance QTL, the F_2_‐derived progeny of W12‐25/HJX74 were analysed. The relative seed setting rate of CSSL HC3‐1 was found to be significantly higher (54.1%) than that of HJX74 (43.0%) (Figure 1b). HC3‐1 carried three substitution segments (Figure 1a). Among 175 random plants from the F_2_ population of HC3‐1/HJX74 identified for CTF, the results indicated two QTLs for cold tolerance in HC3‐1, both of which are recessive or semi‐dominance for cold tolerance (Figure 1c–e). Further analysis confirmed two cold tolerance QTLs, *qCTF3* and *qCTF6* on chromosomes 3 and 6 based on single segment substitution line (SSSL) (Figures 1f and S4). Both QTLs showed increased relative seed setting rate, with additive effects of 3.8% and 3.9%, respectively (Figure 1f). *qCTF3* with an interval length of 2.1 Mb on chromosome 3 (S20‐192‐27), while *qCTF6* was mapped to a 0.1 Mb interval on chromosome 6 (S20‐9‐18) (Figure S4).

To assess the breeding applications value of *qCTF6*, we examined 11 agronomic traits between HJX74 and the SSSL S20‐9‐18 during the first crop season (February to July) of 2021. Nine agronomic traits did not differ significantly between HJX74 and S20‐9‐18. However, two agronomic traits, taste score and thousand grain weight, showed highly significant differences between HJX74 and S20‐9‐18 (Figure S5).

### Candidate genes analysis of *qCTF6*

**Figure 2:**
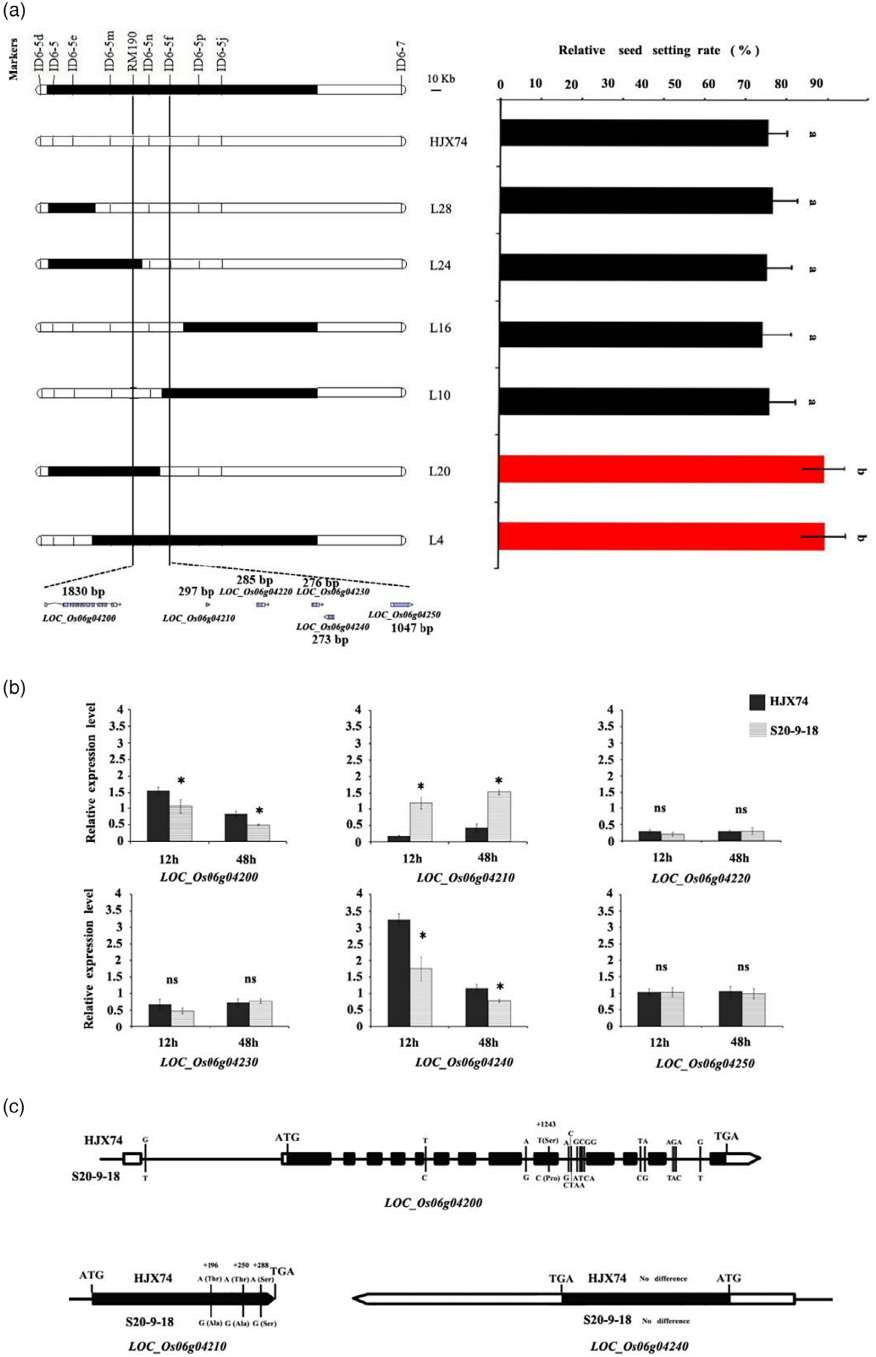
Candidate gene analysis of *qCTF6*. (a) Substitution mapping of *qCTF6*. White and black blocks on chromosomes represent genotypes of HJX74 and IR58025B, respectively. Different alphabet letters denote differences at 0.05 level of significance in Duncan test. The values of relative seed setting rate are presented using mean and standard deviations; (b) the expression level of six candidate genes measured by qRT‐PCR. * indicates significance of difference at *P* < 0.05 based on t‐test, ns means no significant difference; (c) the sequence variations of three genes between HJX74 and S20‐9‐18. Black boxes indicate exons, white boxes indicate 5′ and 3′ UTRs.

Fine mapping of *qCTF6* was carried out due to its stable cold tolerance effect, promising breeding applications value and small putative interval. Six secondary SSSLs were evaluated for CTF and substitution mapping showed that *qCTF6* was delimited to the RM190‐‐ID6‐5f interval, which is about 28 kb and contained six putative candidate genes (Figure 2a). Among the six putative candidate genes, three candidate genes, *LOC_Os06g04200*, *LOC_Os06g04210* and *LOC_Os06g04240* were significantly differentially expressed in HJX74 and S20‐9‐18 after cold stress (Figure 2b). The expression levels of *LOC_Os06g04200* and *LOC_Os06g04240* in S20‐9‐18 were significantly lower than that in HJX74; whereas the expression of *LOC_Os06g04210* in S20‐9‐18 was significantly higher than that in HJX74.

These three differentially expressed genes (Figure 2c) and three non‐differentially expressed genes (Figure S6) were compared for genetic variations in the coding DNA sequence (CDS) of W12‐25 (S20‐9‐18 is its derived line) and HJX74. Among the three genes with expression differences, *LOC_Os06g04200* has a non‐synonymous SNP difference (T‐C) at +1243 site; *LOC_Os06g04210* has two non‐synonymous coding variants (A‐G) at +196 and + 250 sites. *LOC_Os06g04240* has no differences in the CDS region (Figure 2c).

Take together, *LOC_Os06g04200*, *LOC_Os06g04210* and *LOC_Os06g04240* were considered promising candidate genes underlying *qCTF6*.

### *CTF1* is the causal gene underlying *qCTF6*

**Figure 3:**
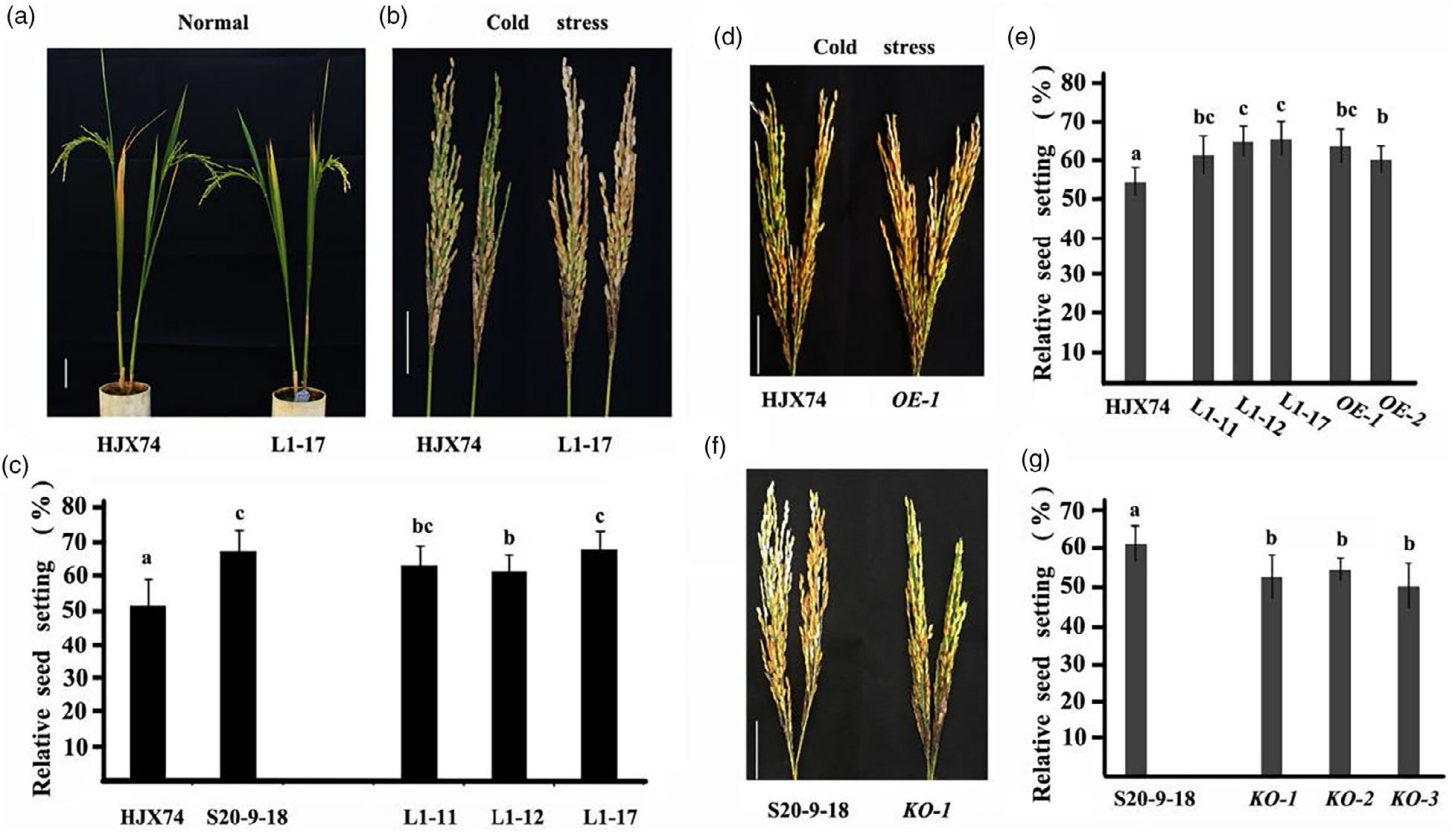
*CTF1* conferring cold tolerance at the flowering stage. (a) HJX74 and the complementary line L1‐17 grown at normal temperature. Scale bar, 10 cm; (b and c) HJX74 showed lower relative seed setting rate than the complementary lines after cold stress; (d and e) HJX74 showed lower relative seed setting rate than the over‐expression lines after cold stress; (f and g) S20‐9‐18 showed higher relative seed setting rate than the knockout lines after cold stress; scale bar, 5 cm. The values of relative seed setting rate are presented using mean and standard deviations. Different alphabet letters denote differences at 0.05 level of significance in Duncan test.

To determine the causal gene underlying *qCTF6*, we constructed complementary vectors of the three candidate genes with the S20‐9‐18 genotype and obtained their transgenic plants in HJX74, respectively. Cold tolerance evaluation showed that only *LOC_Os06g04210* complement transgenic plants showed an improved cold tolerance compared to HJX74 (Figures 3a–c and S7). This suggests that *LOC_Os06g04210* derived from S20‐9‐18 has a semi‐dominant effect for cold tolerance. Further analysis revealed that the relative seed setting rate of *LOC_Os06g04210* over‐expression lines was significantly increased compared to HJX74 after cold treatment (Figures 3d,e and S8a); whereas the relative seed setting rate of *LOC_Os06g04210* knockout lines was significantly decreased compared to S20‐9‐18 (Figures 3f,g and S8b). These results indicate that *LOC_Os06g04210* is the causal gene regulating CTF. As the first reported CTF gene, *LOC_Os06g04210* has been named as *CTF1*. CTF1 is annotated as an expressed protein, a conserved putative protein with 99 amino acids.

### Two SNPs in the CDS cause differences in CTF

**Figure 4:**
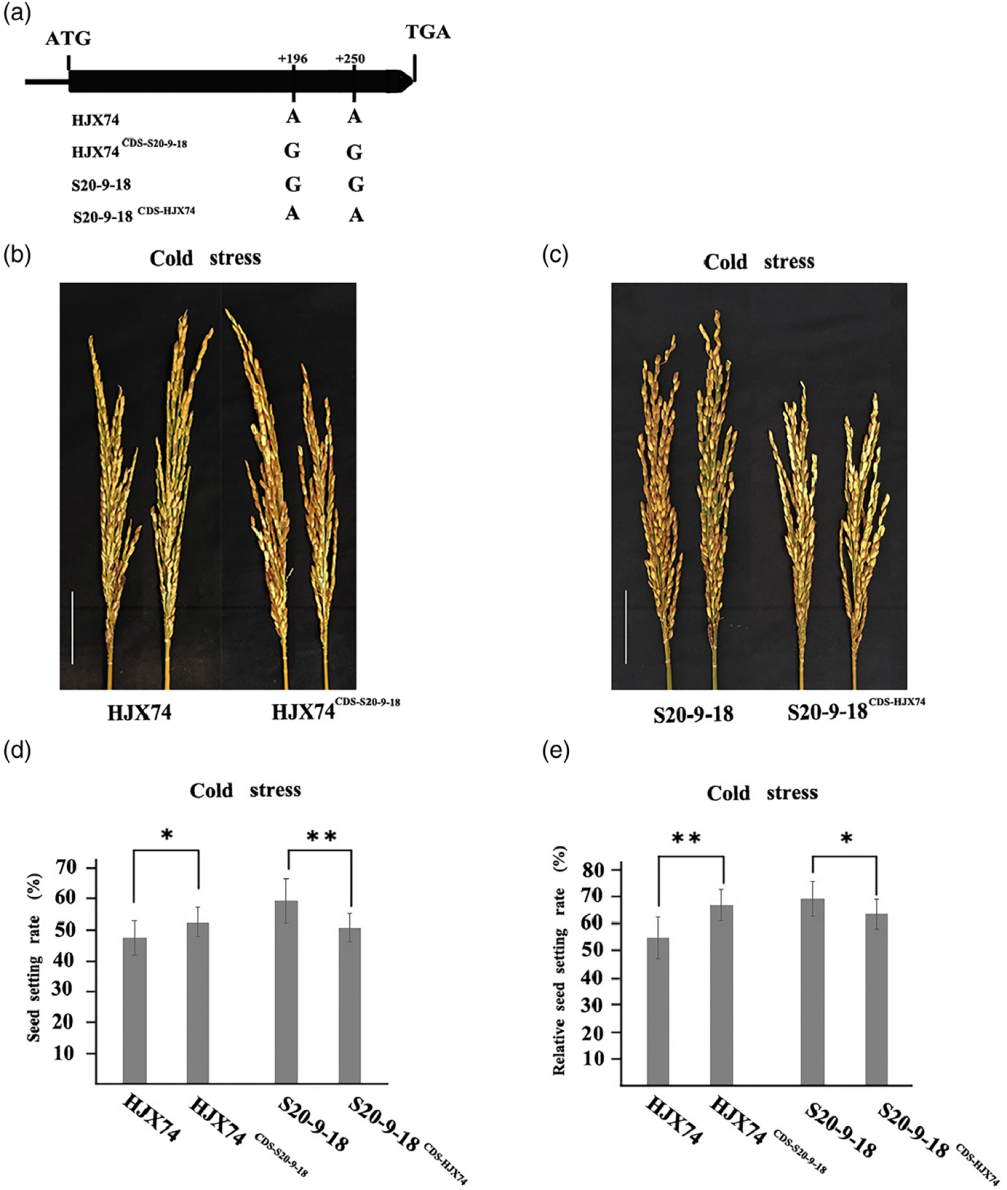
Two SNPs in the CDS region of *CTF1* induces differences in cold tolerance at the flowering stage. (a) Genotypes of control and base substitution lines; (b and c) the performance of seed setting rate of control and base substitution lines after cold stress; the seed setting rates (d) and the relative seed setting rates (e) of control and base substitution lines after cold stress. Scale bar, 5 cm. * and ** indicate significance of difference at *P* < 0.05 and *P* < 0.01, respectively, based on *t*‐test. The values of relative seed setting rate are presented using mean and standard deviations.

Since *CTF1* has two SNPs in the CDS of HJX74 and S20‐9‐18 that lead to amino acid variation, to explore the function of specific SNPs in the ORF of *CTF1*, we used base editing to change the two SNPs by PhieABE/PhieCBE, and got two bases edited transgenic lines, HJX74^CDS‐S20‐9‐18^ and S20‐9‐18^CDS‐HJX74^ (Figure 4a). Under cold stress, HJX74^CDS‐S20‐9‐18^ showed a highly significant increase in seed setting rate as well as relative seed setting rate compared with HJX74 (Figure 4b,d,e), whereas S20‐9‐18^CDS‐HJX74^ showed significant decrease in seed setting rate and relative seed setting rate compared with S20‐9‐18 (Figure 4c–e). This suggests that these two missense mutations in the CDS affect CTF in rice.

### Differences in *CTF1* expression levels lead to variations in CTF

**Figure 5:**
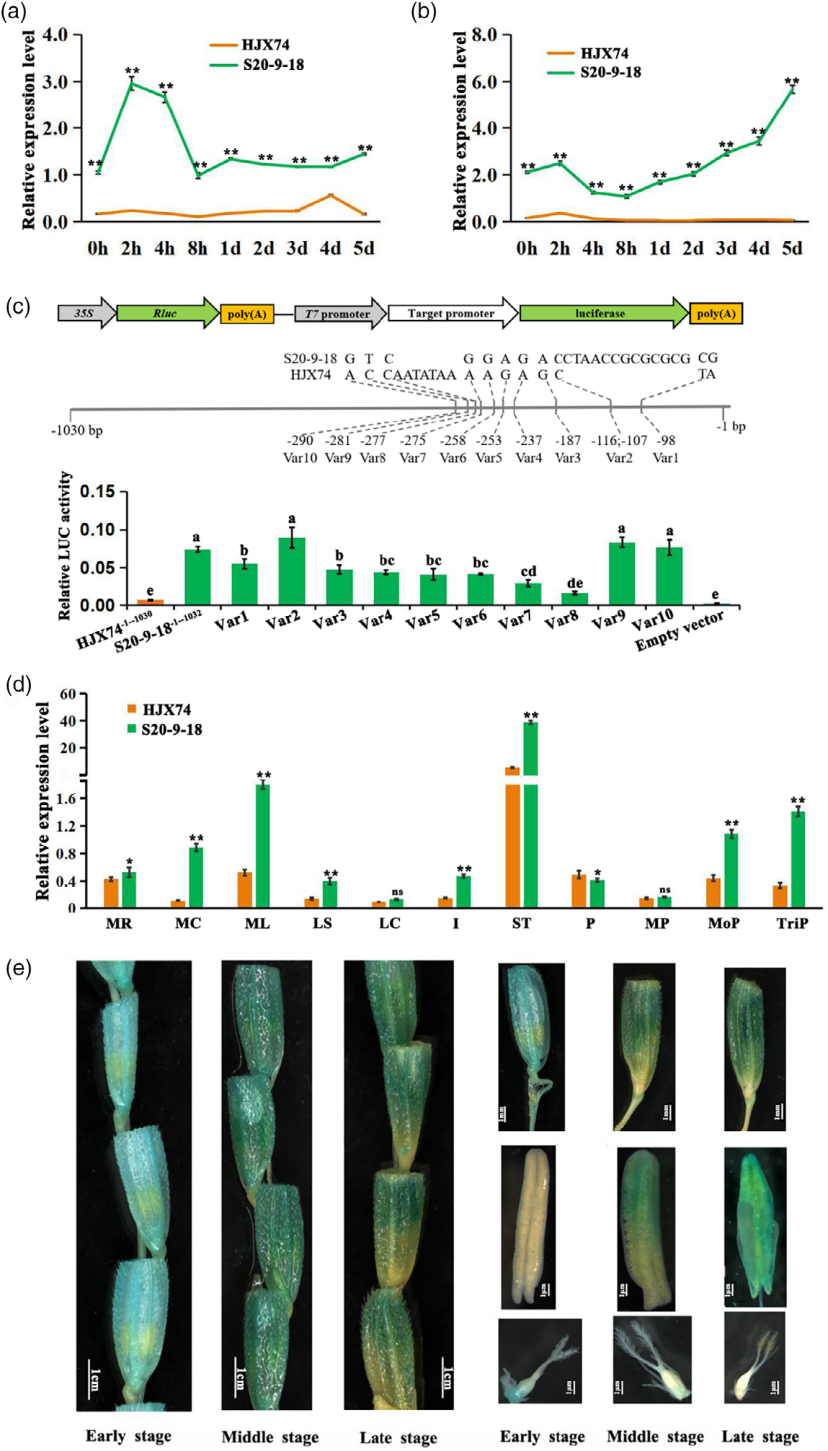
Expression pattern of *CTF1*. (a and b) The expression level of *CTF1* in HJX74 was lower than NIL S20‐9‐18 in both spikelets (a) and flag leaves (b) under cold stress, ** indicates significance of difference at *P* < 0.01 based on *t*‐test; (c) transient expression assay of promoter activity of *CTF1* in rice protoplasts. Top panel, the schematic diagrams of constructs used in the proplast transcription system. Middle panel, the constructs with site‐directed mutations at the 10 variation sites in the S20‐9‐18 type promoter region. Bottom panel, relative LUC values of transcriptional activation activity assays in protoplasts. The empty vector was used as control of fusion constructs. Mean and SD are given by three biological replicates. Different alphabet letters denote differences at 0.05 level of significance in Duncan test; (d) the expression levels of *CTF1* in various tissues of HJX74 and NIL S20‐9‐18. MR: root of mature rice; MC: clum of mature rice; ML: leaf of mature rice; LS: leaf sheath; LC: leaf cushion; I: internode; ST: stamen; P: pistil; MP: panicle at meiosis stage; MoP: panicle at mononuclear stage; TriP: panicle at trinuclear period. * and ** indicates significance of difference at *P* < 0.05 and *P* < 0.01 based on *t*‐test respectively, ns means no significant difference; e. the spikelet GUS staining of *pCTF1* ^*S20‐9‐18*^:: GUS transgenic plants. The GUS staining of spikelets at different development stages (early, middle and late) and their corresponding anthers and pistils.

**Figure 6:**
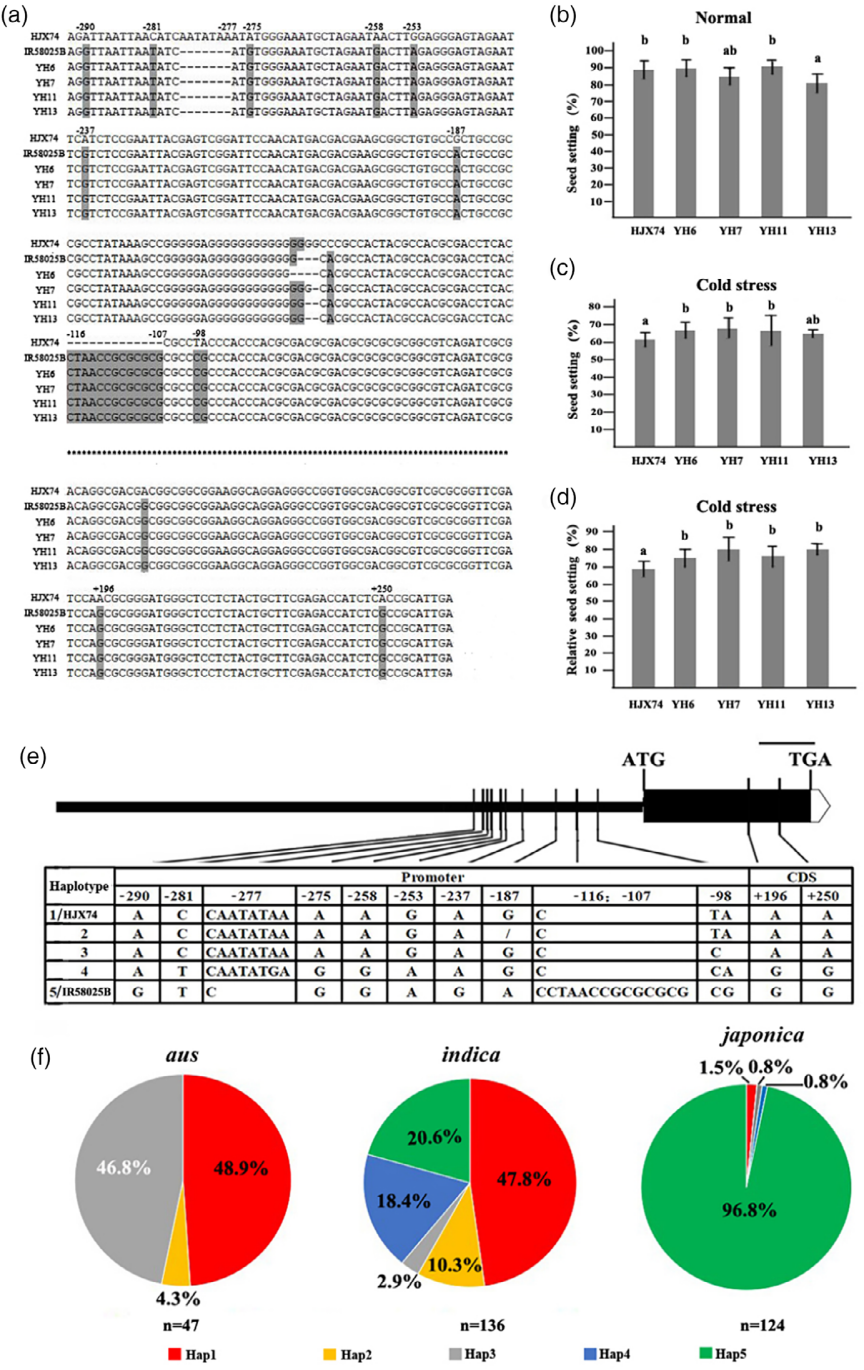
Haplotype analysis of *CTF1*. (a) Sequences of *CTF1* of four cold‐tolerant SSSLs, HJX74 and S20‐9‐18; the seed setting rate of four cold‐tolerant SSSLs and HJX74 grown at normal temperature (b), after cold stress (c), and relative seed setting rate after cold stress (d). Different alphabet letters denote differences at 0.05 level of significance in Duncan test. The values of relative seed setting rate are presented using mean and standard deviations; (e) haplotypes of *CTF1*; scale bar,100bp; (f) the distribution of haplotypes of *CTF1* in three subspecies.

In order to analyse the expression characteristics of *CTF1* under cold stress, the expression levels of *CTF1* in HJX74 and S20‐9‐18 were examined using spikelets and flag leaves. During cold treatment, the expression of *CTF1* in both spikelets and flag leaves of S20‐9‐18 was significantly higher than that of HJX74 (Figure 5a,b). The activities of the *CTF1* promoters from HJX74 and S20‐9‐18 were tested using transient expression assay. The results showed that the activity of promoter sequence (−1 to −1032 bp) from S20‐9‐18 is significantly higher than that of the promoter sequence (−1 to −1030 bp) from HJX74 (Figure 5c). Based on the main ten differences of *CTF1* promoter sequences between HJX74 and S20‐9‐18 (Figure 6e), we synthesised ten mutated sequences based on S20‐9‐18 *CTF1* promoter sequences (Table S4) and conducted transient expression assay. The results indicated that seven variation sites, including Var1, Var3, Var4, Var5, Var6, Var7, and Var8, were functional variation sites that account for the expression difference between HJX74 and S20‐9‐18 (Figure 5c). *CTF1s* of S20‐9‐18^CDS‐HJX74^ and HJX74 contain the same CDS but different promoters. Under cold stress, S20‐9‐18^CDS‐HJX74^ showed a highly significant increase in relative seed setting rate compared with HJX74 (Figure 4e). This suggests that variation in the *CTF1* promoter also contributes to the CTF differences.

Further, the expression of *CTF1* in different tissues revealed that the highest expression of *CTF1* was in stamens, and the expression level in S20‐9‐18 was much higher than that in HJX74 (Figure 5d). GUS staining of spikelets at early, middle and late development stages showed that *CTF1* was highly expressed at the middle and late stages during spikelet development, and mainly in stamens but not pistils (Figure 5e). The specific high expression of *CTF1* in anthers at the middle and late stages of spikelet development further supports the biological function of *CTF1* in regulating CTF.

### Haplotype analysis of *CTF1*

SSSLs with *CTF1* under the genetic background of HJX74 were selected from the SSSL library for CTF evaluation, and four SSSLs from different donors were colder tolerant than HJX74 (*P* < 0.05) (Figure 6b–d). The *CTF1* of these four SSSLs were sequenced and compared. In the promoter region, there were 12 mutations in the four SSSLs compared with HJX74, and 11 mutations remain consistent among the four SSSLs (Figure 6a). In the CDS, there were three mutation sites in the four SSSLs compared to HJX74, with the mutations being A‐G, and two of them were missense (Thr‐Ala) (Figure 6a). The 13 conserved mutations (11 mutations in promoter and two non‐synonymous variants in CDS) in the four SSSLs were also consistent with the sequence of IR58025B (Figure 6a).

To further clarify the haplotypes of *CTF1*, 307 accessions from RDP2 were used for haplotype analysis based on these 13 sites. There were five haplotypes in the whole population (Figure 6e); Hap1/Hap‐HJX74 was the cold‐sensitive haplotype, and Hap5/Hap‐IR58025B was the cold‐tolerant haplotype. In the *aus*, there were three haplotypes (Hap1‐3), with Hap1/Hap‐HJX74 and Hap3 accounting for 48.9% and 46.8%, respectively. In *indica*, there were five haplotypes (Hap1‐5), with the highest haplotype frequency being Hap1/Hap‐HJX74 with 47.8%. In *japonica*, there were four haplotypes (Hap1, 3, 4 and 5), and the highest haplotype frequency was Hap5/Hap‐IR58025B with 96.8% (Figure 6f).

### Positive selection of *CTF1* contributed to the cold adaptation of *japonica*

**Figure 7:**
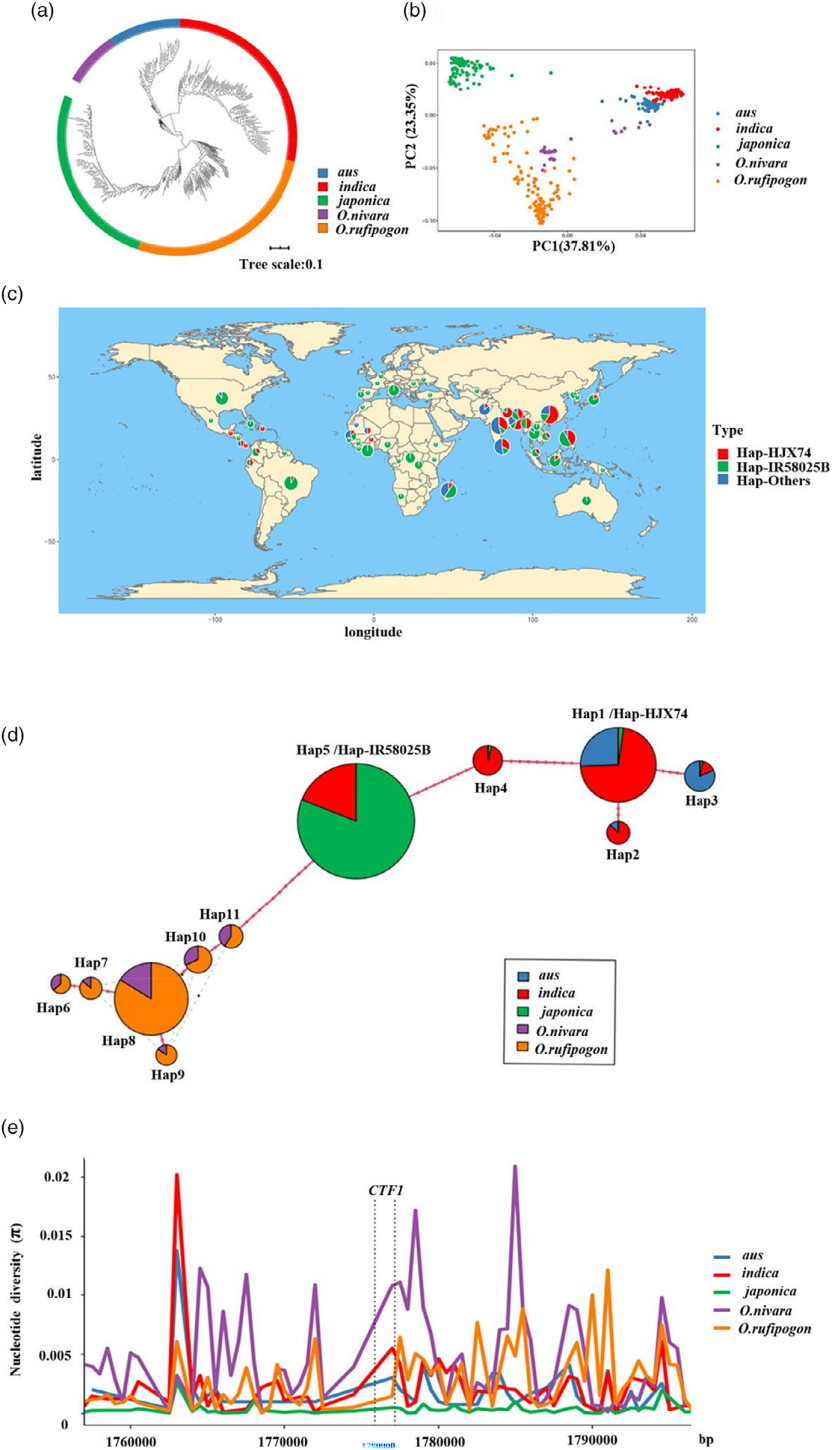
Phylogenetic and population genetic analysis of *CTF1* in rice. (a and b) Phylogenetic tree and principal component analysis of 472 accessions; (c) geographic distributions among 307 accessions. The Hap‐HJX74 and Hap‐IR58025B members are indicated by red and green, respectively; other accessions is indicated by blue; (d) haplotype network of *CTF1*. Circle size is proportional to the number of samples for a given haplotype. Black spots represent unobserved, but inferred haplotypes. Lines between haplotypes represent mutational steps between alleles; (e) nucleotide diversity of *CTF1* in sRDP2 populations and the wild rice group. The *X*‐axis denotes the position of *CTF1* and the *Y*‐axis indicates average *π* values.

472 accessions with diverse genetic diversity including 165 wild rice lines (36 *O. nivara* and 129 *O. rufipogon*) and 307 accessions from RDP2 (Table S2) were employed to investigate the geographic and phylogenetic origins analysis of *CTF1* (Figure 7a,b). To examine the geographic distribution of the haplotypes, we classified the 307 accessions into three haplotypes, namely Hap1/Hap‐HJX74, Hap5/Hap‐IR58025B, and Hap‐others. The distribution of accessions with Hap1/Hap‐HJX74 and Hap5/Hap‐IR58025B shows significant geographical characteristics. Among the 307 accessions, Hap1/Hap‐HJX74 is present in 3.3% of those from 3 to 26° S and 2.2% from 30 to 51° N. Hap5/Hap‐IR58025B is found in 21.6% of accessions from 3 to 26° S and 22.3% from 30 to 51° N (Figure 7c). Hap5/Hap‐IR58025B has a higher frequency distribution at higher latitudes than Hap1/Hap‐HJX74. Moreover, the phylogenetic analysis of *CTF1* (1 kb upstream and CDS) based on 13 sites in 472 accessions showed that there were six novel *CTF1* haplotypes (Hap6‐11) among the 165 wild rice (36 *O. nivara* and 129 *O. rufipogon*), none of which were identical to the five haplotypes in the 307 rice accessions (Figure S9). The Hap5/Hap‐IR58025B was absent in wild rice but account for 96.8% in *japonica* (Figures 6f and 7d). These results imply that Hap5/Hap‐IR58025B does not directly originate from *O. nivara* or *O. rufipogon* but was selected in *japonica*. Since 96.8% of *japonica* contained Hap5/HAP‐IR58025B and 20.6% of *indica* also contained Hap5/HAP‐IR58025B, this implied that *CTF1* might infiltrate from *japonica* to *indica*. Therefore, four‐taxon D statistic were performed, and the results showed that there was an infiltration signal of *CTF1* between *japonica* and *indica* (Figure S10). We analysed nucleotide diversity within the *CTF1* and across a 20 kb upstream and downstream region. This analysis included samples from *aus*, *indica*, *japonica*, *O. nivara*, and *O. rufipogo*n to ascertain whether selective pressures have influenced the selection of *CTF1* (Figure 7e, Table S5). On average, the nucleotide diversity of *CTF1* in *O. nivara* (*π* = 0.01083), *indica* (*π* = 0.0041), *aus* (*π* = 0.002), *O. rufipogon* (*π* = 0.001) was higher than in *japonica* (*π* = 0.00035) (Table S5). The fixation index (Fst) was calculated between *japonica* and other subpopulations, revealing relatively high genetic differentiation in *japonica* compared to other populations within the genomic regions of *CTF1* (Figure S11). Neutrality tests were also conducted, revealing a Tajima's *D* value of −2.02 (*P* < 0.05) in *japonica* (Table S6). Taken together, these findings collectively indicate that *CTF1* is positively selected to cold adaptation in *japonica*.

## Discussion

### *qCTF3* and *qCTF6* are novel QTLs with stable effects for CTF

Cold tolerance at the reproductive stage (CTR) in rice is a complex trait controlled by multiple QTLs and significantly influenced by environmental factors (Tang *et al*., 2019; Yang *et al*., 2019). The stability of QTLs associated with CTR across various genetic backgrounds and environments remains largely unconfirmed. Given the intricate nature of field conditions, multi‐year QTL assessments are imperative for pinpointing those that substantially impact breeding for cold tolerance. These stably expressed QTLs will play an important role in molecular breeding for rice cold tolerance.

This study sought to identify QTLs that could effectively contribute to breeding for rice cold tolerance. To this end, we employed the single segment substitution line (SSSL) population constructed using IR58025B, known for good CTF, and HJX74, a popular variety in Southern China. From this, we selected the chromosome segment substitution line (CSSL) W12‐25, which exhibited stable CTF and a higher relative seed setting rate than HJX74 after cold treatment in three independent experiments. Furthermore, CSSL HC3‐1, derived from crossing W12‐25 and HJX74, demonstrated promising CTF in two independent tests (Figure 1b). Moreover, the SSSL and secondary SSSLs (carrying the cold tolerance QTL *qCTF3* or *qCTF6*) derived from crossing HC3‐1 and HJX74 both exhibited higher relative seed setting rates than HJX74 after cold treatment (Figure 1f). Cold tolerance was stably detected in multiple generations of experimental material over many years, demonstrating that *qCTF3* and *qCTF6* are stable QTL for CTF.

The *qCTF3* contains the cold tolerance gene *OsAPX1* (Sato *et al*., 2011) and *OsMAPK3* (Lou *et al*., 2022). The *qCTF6* partially overlaps with the QTLs *qRCT6a* (Dai *et al*., 2004), *qCT6.1* (Liang *et al*., 2018) and *MCqtl6‐1* (Yang *et al*., 2019). These results indicate that *qCTF3* and *qCTF6* are stably expressed at the reproductive stage under different genetic backgrounds and low temperatures and have promising breeding potential.

Four genes of CTB were identified by map‐based cloning, *Ctb1* (Saito *et al*., 2010), *CTB4a* (Zhang *et al*., 2017), *LTT1* (Xu *et al*., 2020) and *COG3* (Liu *et al*., 2024). Three of these cold tolerance genes were derived from *japonica* and one was a mutation; no cold tolerance genes have been reported from *indica*. The cold tolerance QTLs from *japonica* have limited use in *indica* breeding due to intersubspecific hybrid sterility (Ouyang *et al*., 2010). However, in this study, both *qCTF3* and *qCTF6* significantly enhanced CTF, were derived from *indica* IR58025B. This allows us to avoid intersubspecific hybrid sterility, which will significantly enhance cold‐tolerant *indica* breeding.

### *qCTF6* has promising breeding potential

Molecular breeding primarily employs molecular markers to introduce targeted QTL into improved lines once they have been mapped. This process aims to develop new lines exhibiting enhanced target traits. However, linkage drag may co‐introduce unfavourable agronomic traits alongside the improvement of the target trait, posing a conundrum for breeders (Wang *et al*., 2020; Xiao *et al*., 2021). This issue may sometimes lead breeders to forsake the application of certain QTLs to maintain the balance of agronomic traits. As such, QTLs devoid of linkage drag are widely useful in molecular breeding.

To assess the breeding potential of *qCTF6*, we conducted a field assessment of 11 major agronomic traits between HJX74 and S20‐9‐18 carrying *qCTF6*. There were no significant differences in nine agronomic traits (Figure S5), except for taste score and thousand grain weight. The taste score of S20‐9‐18 was 14.5 points higher than that of HJX74, indicating a marked improvement in eating quality, without any significant alteration in grain size. This improvement is likely attributed to the presence of the *Wx* (*LOC_Os06g04200*) within the *qCTF6* region, a key determinant of amylose synthesis and thus rice eating quality. *Wx* from HJX74 confers a high amylose content (24.58%–25.20%), while *Wx* from IR58025B results in a middle amylose content (12.34%–15.63%) (Teng *et al*., 2012). Given the paramount importance of eating quality in modern rice production and its impact on market value, the *qCTF6* not only enhances this quality but also confers improved seed setting rates under low‐temperature conditions at the flowering stage. These dual benefits align with current rice breeding objectives and underscore the potential of *qCTF6* in advancing rice production.

The linkage molecular markers developed in the *qCTF6* interval will facilitate the use of IR58025B as a source of high‐quality and cold‐tolerant QTLs to breed superior new varieties. S20‐9‐18 carries only a 100 kb chromosome segment derived from IR58025B in the genetic background of the Guangdong *indica* cultivar HJX74, making it a promising new breeding line.

### *CTF1* regulates CTF through genetic variations of promoter and coding region in rice

Elucidating the genetic basis of natural variation in cold tolerance and cloning the corresponding genes is crucial for enhancing the cold tolerance of crops. CTR is particularly critical, as it significantly affects grain yield and quality (Espe *et al*., 2017; Zhang *et al*., 2014). To our knowledge, this is the first cloned and functionally confirmed gene for CTF in rice.

Variation in gene expression or/and amino acids are recognised as primary sources of functional divergence among genes. The emergence of high expression levels of favourable genes under low‐temperature stress, thus showing cold tolerance, is a distinctive feature of cold‐tolerant genes. Previously reported cold‐tolerant genes, such as *OsMAPK3*, *CTB4a*, *qCTB7* and *COG3* show significantly higher expression levels in cold‐tolerant lines than in cold‐sensitive lines during cold treatment (Liu *et al*., 2024; Lou *et al*., 2022; Yang *et al*., 2023a; Zhang *et al*., 2017). In this study, *CTF1* expression in both spikelets and leaves of cold‐tolerant NIL S20‐9‐18 was significantly higher than that of HJX74 under cold treatment (Figure 5a,b). Additionally, the promoter activity of *CTF1* ^*S20‐9‐18*^ was found to be higher than that of *CTF1* ^*HJX74*^ (Figure 5c). Furthermore, seven functional variation sites were found in the promoter region. S20‐9‐18^CDS‐HJX74^ showed a highly significant increase in relative seed setting rate compared with HJX74 under cold stress (Figure 4e). These results indicate that the promoter variation in *CTF1* contributes to the differential cold tolerance.

Differences in amino acids caused by base variants in the CDS may also be a primary reason for differences in cold tolerance of genes, such as *COLD1* and *CTB2* (Li *et al*., 2021b; Ma *et al*., 2015b). These differences arise from base variants in the coding region, resulting in differences in cold tolerance. In this study, two SNPs in the CDS of *CTF1* that result in amino acid changes were identified, and their impact on CTF was confirmed by single‐base edited transgenic lines (Figure 4). It is relatively rare to validate the functional regions of genes by single base edit in rice.

In summary, *CTF1* is the first gene demonstrated to regulate cold tolerance through variations in both the promoter and CDS. Comparative analysis of *CTF1* across 307 accessions revealed five distinct haplotypes (Figure 6e). Hap5/Hap‐IR58025B is a cold‐tolerant haplotype, and compared with the other four haplotypes, has significant sequence features at two insertions/deletions in the promoter region (Figure 6e), allowing for the design of intragenic molecular markers to accurately select the favourable haplotype. The identification of these haplotypes and molecular markers suggests that *CTF1* can be effectively utilised to improve cold tolerance in rice through molecular breeding.

To further investigate the molecular mechanism of *CTF1* in regulation of CTF, RNA sequencing was conducted on HJX74 and its complementary line L1‐17. After cold stress, 129 genes exhibited differential expression in HJX74 and L1‐17, including *CTF1* (Figure S12a). Of the 11 causal genes that have been reported to be expressed at the booting stage, the expression levels of nine genes did not differ significantly between HJX74 and L1‐17, while two genes were not expressed (Figure S12b). This implies that *CTF1* employs a distinct regulatory mechanism for cold tolerance compared to these genes. The GO enrichment analysis of the differentially expressed genes revealed that the up‐regulated gene set was significantly enriched in processes related to terpenoid biosynthesis and metabolism, particularly in diterpenoid. Conversely, the down‐regulated genes demonstrated significant enrichment in several fundamental metabolic pathways, including responses to copper ions, the tricarboxylic acid metabolic process, and lipid binding (Figure S12c). These findings provide clues to reveal the molecular mechanism by which *CTF1* leads to differences in CTF.

### The cold‐tolerant haplotype of *CTF1* was generated by natural variation and was positively selected for in *japonica*

The process of domestication necessitates the acquisition of favourable haplotypes that enhance adaptation to environmental challenges. These beneficial haplotypes can arise from ancestral standing variation or through newly generated mutations (Barrett and Schluter, 2008).

The currently reported genes, *Ctb1* (Saito *et al*., 2010), *OsAPX1* (Sato *et al*., 2011), *CTB4a* (Zhang *et al*., 2017), *bZIP73* (Liu *et al*., 2019), *LTT1* (Xu *et al*., 2020), *CTB2* (Li *et al*., 2021), *OsMAPK3* (Lou *et al*., 2022), *OsLEA9* ^*KL*^ (Lou *et al*., 2022), *WRKY53* (Tang *et al*., 2022), *qCTB7* (Yang *et al*., 2023a) and *COG3* (Liu *et al*., 2024) have been shown to significantly enhance CTB. *OsMAPK3*, *CTB2* and *COG3* directly evolved from *O. rufipogon*, which was mainly retained in *japonica* during rice domestication (Li *et al*., 2021b; Liu *et al*., 2024; Lou *et al*., 2022). *CTB4a* and *OsLEA9* ^*KL*^ originated from a novel mutation in *japonica* accessions under cold climatic conditions (Lou *et al*., 2022; Zhang *et al*., 2017). In this study, *CTF1* conferred CTF in rice. Accessions carrying the cold‐tolerant haplotype of *CTF1* (Hap5/Hap‐IR58025B) were more widely distributed than those carrying other haplotypes (Figure 7c); haplotype analysis revealed that, although as many as six haplotypes existed in wild rice, none was the Hap5/Hap‐IR58025B (Figure 7d), which imply that Hap5/Hap‐IR58025B does not directly originate from *O. rufipogon* or *O. nivara* during *japonica* domestication. In *japonica*, the sharp decrease in nucleotide diversity in *CTF1* and its adjacent intervals strongly suggests that it may have been subjected to positive selection (Figure 7e). Therefore, we suggest that during the domestication of wild rice into *japonica*, *CTF1* generated novel mutations for cold acclimation and was subsequently selected in *japonica* to adapt to the cooler climates of the cultivated regions. Interestingly, the Hap5/Hap‐IR58025B has not been widely distributed in *indica* and thus has great potential for genetic improvement of CTF in *indica* (Figure 6f). Molecular breeding using the *CTF1* ^*Hap5/Hap‐IR58025B*^ will further enhance the cold tolerance of *indica* cultivars, minimising yield loss caused by extreme cold weather at the flowering stage.

## Materials and methods

### Materials

Twenty‐one SSSLs derived from *indica* IR58025B as donor and *indica* HJX74 as recipient and their parents (Zhang, 2021; Zhang *et al*., 2004) were used to evaluate CTF. The procedure for the developing of these SSSLs was as follows. Since 1998, IR58025B has been crossed with the recipient HJX74. The F_1_ hybrids were then backcrossed to HJX74. The cross was screened with 300–400 polymorphic SSR markers evenly covering the entire rice genome to identify substitution segments and the genetic background in the segregating population. After backcrossing 3–7 times with marker‐assisted selection, plants carrying single substitution segments from the donor IR58025B in the recipient genetic background were selected. Homozygous SSSLs were then developed by selfing (Zhang, 2021). The SSR markers location reference McCouch *et al*. (2002).

Combined with molecular marker‐assisted selection, nine SSSLs were obtained by backcrossing the cold‐tolerant chromosome segment substitution line (CSSL) W12‐25 with HJX74. Six secondary SSSLs were obtained from the cold‐tolerant SSSL S20‐9‐18. The development of the experimental material is shown in Figure S1.

The four cold tolerance SSSLs with *CTF1* used for haplotype analysis were derived from SSSL library (Zhang *et al*., 2004), and the detail were list in Table S1. The 307 accessions from RDP2 and 165 wild rice used for haplotype anlaysis of *CTF1* were list in Table S2.

### Evaluation of CTF

The accessions for the test was grown as a single plant in 11.0 cm diameter × 23.0 cm high buckets filled with soil and placed in a net room with regular water and fertiliser management. Tillers were removed during plant growth, leaving only the main panicle and a maximum tiller panicle, following the method of Andaya and Mackill (2003). At the heading date, individual plants of the same heading date were randomly divided into two groups of 15 plants each and placed in the CONVIRON PGV36 climate chamber set at 28.0°C/25.0°C (control), 15.6°C (treatment, Figure S2), 70% relative humidity and a 12 h light/12 h dark cycle, a light intensity of 10 000 lux. After 7 days treatment, the plants were returned to the net room for further growth. At maturity, the seed setting rate of individual plants was measured and the relative seed setting rate was used to measure the CTF in tested lines. Relative seed setting rate is calculated as seed setting rate after cold treatment/seed setting rate of control × 100%.

### Determination of important agronomic traits

The lines to be tested were planted in early season (February to July) in 2021 at the Guangzhou experiment station. The SSSL and HJX74 were sown to the seedbed on 1 March and transplanted to the field on 30 March, single planted with a spacing of 19.6 cm × 19.6 cm and managed with conventional water and fertiliser. Three plots were planted for each accession, with 40 plants in each plot, and 20 plants that were uniform in the centre of the plot were taken to examine traits. Eight traits were tested including heading date, plant height, number of effective spikes per plant, thousand grain weight, seed setting rate, grain number per panicle, grain yield per plant and harvest index according to the Standard Evaluation Systems for Rice (IRRI, 1996). The grain length and grain width of each line were determined by using a Wanshen automatic grain analyser (Hangzhou Wanshen Testing Technology Co., Ltd., Zhejiang, China). The taste score of rice is measured by rice taste measuring device (SATAKE corporation, Japan).

### Statistics and QTL analysis

As the SSSL differs from the recipient HJX74 by only one chromosomal substitution segment, all heritable phenotypic variations are associated with this substitution segment. Therefore, once the relative seed setting rate of SSSL differed significantly from that of HJX74, a QTL for cold tolerance or sensitivity was identified in this SSSL. Additive effects of the QTL were estimated by the method of Eshed and Zamir (1995), additive effect value = (phenotypic value of the QTL – the phenotypic value of HJX74)/2. The naming of QTLs followed the rules proposed by McCouch *et al*. (1997). Student's two‐tailed *t* test was used for significant difference analysis between the two samples. One‐way ANOVA analyses followed by Duncan's test or *LSD* test (*P* < 0.05) were used for multiple comparisons. Substitution mapping was performed according to the method of Tan *et al*. (2021). If the same QTL was detected in multiple SSSLs, then this QTL was located in the overlapping interval of these SSSLs. If the QTL is detected in only one SSSL and not in other SSSLs that have partial segment overlap with this SSSL, then this QTL is located in the non‐overlapping interval.

### Genotyping of molecular markers

The DNA samples were amplifed by PCR method. PCR amplification and band type detection were performed according to the method of Yang *et al*. (2016). 15 μL of the reaction system contained 0.15 μmol/L primer, 200 μmol/L dNTP, 1 × PCR reaction buffer (50 mmol/L KCl, 10 mmol/L Tris–HCl, pH 8.3, 1.5 mmol/L MgCl_2_, 0.01% gelatin), 50–100 ng DNA template, and 1 U Taqase. The PCR reactions were performed in a S1000 DNA amplifier (Bio‐Rad, USA) with the following reaction procedure: pre‐denaturation at 94 °C for 5 min; 35 cycles of 94 °C for 30 s, 55 °C for 30 s, and 72 °C for 40 s; and extension at 72 °C for 8 min. The amplification products were separated by 8.0% polyacrylamide gel electrophoresis, stained with Goldview and the band patterns were detected using a gel imaging system (Bio‐Rad, USA). The primer sequences for substitution segment screening and genetic analysis were listed in Table S3.

### DNA re‐sequencing and analysis of variant loci

The leaves of rice seedlings were collected and subjected to DNA extraction by the CTAB method. Sequencing was performed on the Illumina NovaSeq6000 platform for re‐sequencing and Pacbio Sequel II platform for Pacbio sequencing. Sequencing data analysis was conducted using pipeline in our previous study (Yang *et al*., 2023b). Briefly, fastx_toolkit (http://hannonlab.cshl.edu/fastx_toolkit) was used to remove adaptor and low‐quality reads. Short read re‐sequencing data were aligned to the reference genome using BWA‐MEM, only reads with a perfect match or one mismatch were further analysed and annotated based on the Nipponbare IRGSP1.0 genome. Then nucleotide variants for each accession were detected and filtered using GATK (v3.8‐1‐0) with the default parameters. All reads have been deposited in the NCBI sequence read archive (BioProject accession PRJNA985385).

### Genome assembly of W12‐25 and analysis of variant loci

Genome assembly of W12‐25 was conducted using the pipeline in our previous study (Wang *et al*., 2023). Briefly, The Pacbio raw data was implemented to *de novo* assemble with the software MECAT and polished using the 100× Illumina reads by NextPloish. RaGOO was then used to order the assembly contigs. Space between contigs was artificially filled in with 100 “N” blocks. The assembled W12‐25 genome sequence was used to identify sequence variants by blast with the HJX74 genome sequence (Li *et al*., 2021a ).

### Expression analysis of candidate genes

Total RNA was extracted from panicles and other tissues using Trizol reagent (Invitrogen, Carlsbad, CA, USA) and purified using RNeasy Plant Mini Kit (Qiagen, Valencia, CA). 1 μg of total RNA was taken and reverse transcribed using AMV (TAKARA); the primers for qRT‐PCR were designed by Primer Premier 5.0 and real‐time PCR was carried out using the SYBR Premix ExTaqTM kit (Takara) on a Biorad CFX 96 Real‐Time System (Bio‐Rad); fluorescence was detected for quantitative analysis and *OsEF1A* was used as an internal reference. All reactions were run in triplicate. Primers used to amplify the selected genes were listed in Table S3.

### RNA sequencing and analysis

To further investigate the molecular mechanisms underlying *CTF1*, RNA sequencing (RNA‐seq) was conducted to compare gene expression variations between the HJX74 and its complementary line L1‐17. After subjecting the samples to cold stress 1 day, three accessions were selected for detailed analysis. Spikelets from each accession were ground, and RNA extraction was performed using the Trizol reagent (Invitrogen, Carlsbad, CA, USA) and purified using RNeasy Plant Mini Kit (Qiagen, Valencia, CA). A total of six 150 bp paired‐end libraries were constructed and sequenced. The short reads generated from this process underwent quality control and trimming using fastp. The clean reads were then mapped to the Nipponbare genome (IRGSP1.0) using the HiSat2, with only uniquely mapped reads retained for quantifying gene expression via feature Counts. Differential expression analyses were performed using DEseq2 (Love *et al*., 2014), setting a false discovery rate (FDR) threshold of 0.05 and a log2 fold change (FC) cut‐off of 0.5. Additionally, Gene Ontology (GO) functional hypergeometric tests were employed with the analysis carried out using the R package clusterProfiler (Yu *et al*., 2012).

### Vector construction and genetic transformation for candidate genes

For construction of complementation plasmids, a 6654‐bp DNA fragment containing the 2197‐bp natural promoter, full 3480‐bp exon and intron regions, and 977‐bp 3′UTR of *LOC_Os06g04200*; a 1328‐bp DNA fragment containing the 1031‐bp natural promoter and 297‐bp ORF of *LOC_Os06g04210*; a 3689‐bp DNA fragment containing the 2173‐bp natural promoter, 273‐bp ORF, 1243‐bp 3′UTR and downstream region of *LOC_Os06g04240* were amplified from SSSL S20‐9‐18, respectively. Using homologous recombination, the DNA fragments were cloned into binary vector pCAMBIA 1300. For construction of *LOC_Os06g04210* (*CTF1*) overexpression plasmid, the full coding sequence was amplified from the cDNA of S20‐9‐18 and digested with *Hind* III and *Spe* I, then cloned into binary vector pOX driven by Ubiquitin promoter (Peng *et al*., 2023). For construction of gene knock‐out plasmid, two sgRNAs targeted the *CTF1* were designed and assembled into the pYLCRISPR/Cas9 binary vector using Golden‐Gate cloning, as previously described (Ma *et al*., 2015a). All fragments were amplified by the high fidelity PCR enzyme KOD‐FX (TOYOBO, KFX‐101). Primer sequences for vector constructions are provided in Table S3. All plasmids confirmed by sequence were introduced into *Agrobacterium tumefaciens* strain EHA105 and transferred into recipient materials by *Agrobacterium*‐mediated method. The recipient for complementation and overexpression analyses were HJX74. The recipient for gene knock‐out analysis was S20‐9‐18. Homozygous T_2_ lines were verified using hygromycin selection and PCR analysis.

### Base editing for determination of functional SNPs

To explore the function of specific SNPs in the ORF of *CTF1*, we used base editing to change the SNPs as previously reported (Tan *et al*., 2022; Zeng *et al*., 2020). For HJX74, two sgRNA expression cassettes (AG‐U6aT1 and AG‐U6bT2) were used to generate A196G and A250G in the *CTF1*, respectively, mediated by the PhieABE editing. Then the constructs were transformed into the HJX74 by *Agrobacterium*‐mediated transformation with strain EHA105. Similarly, two sgRNA expression cassettes (GA‐U6aT1 and GA‐U6bT2) were used to generate G196A and G250A in the *CTF1*, respectively, mediated by the PhieCBE editing. The constructs were transformed into the NIL S20‐9‐18 by *Agrobacterium*‐mediated transformation with strain EHA105 as well. Genotyping of *CTF1* was performed using primers listed in Table S3.

### GUS staining analysis

For construct *pCTF1:GUS*, a 1031 bp genomic fragment preceding the ATG start codon of *CTF1* was amplified from DNA of NIL S20‐9‐18 using the primers listed in Table S3. After subcloning and sequencing, the correct gene fragments were inserted into pCAMBIA 1300‐GUS vector by *Hind* III and *Xba* I. The positive plasmids were electroporated into *Agrobacterium tumefaciens* EHA105, then introduced into calli of the NIL S20‐9‐18 via *Agrobacterium*‐mediated genetic transformation respectively. The GUS staining was performed as described using 5‐bromo‐4‐chloro‐3‐indolylb‐Dglucuronicacid (X‐Gluc) (Dong *et al*., 2021). Briefly, we incubated the transgenic panicles overnight at 37 °C in staining buffer (100 mM sodium phosphate [pH 7.0], 10 mM EDTA, 0.5 mM K_4_Fe(CN)_6_, 0.5 mm K_3_Fe(CN)_6_, 0.1% [v/v] Triton X‐100 and 1 mM X‐Gluc) and then decolorised in 100% ethanol. Staining was observed using a stereo microscope (Zeiss SteREO Lumar V12).

### Transcriptional activity assays

Transcriptional activity assays were performed as previous reported study (Liu *et al*., 2024). To compare the activity of *CTF1* promoters, promoter sequences (−1 to −1030 bp for HJX74; −1 to −1032 bp for S20‐9‐18) were amplified from the DNA. The 10 mutated *CTF1* promoter sequences (Var1‐10) based on S20‐9‐18 DNA sequences were synthesised by sangon (https://store.sangon.com/). Using homologous recombination, all the DNA fragments were separately cloned into pGreen II‐0800‐Luc vector which with 35S::REN as an internal control. All the vectors were transfected into rice protoplasts using transient transformation (Chen *et al*., 2023). Protoplasts were cultured at 28 °C for 16–18 h in the dark to detect the transcriptional activity under normal condition. The LUC and REN activity were measured using GloMax® 20/20 Luminometer System (Promega), and relative LUC activity was calculated (LUC/REN) to determine relative promoter activity. Three biological replicates, each with three technical replicates, were assayed for each construct. One‐way ANOVA analyses followed by Duncan's test (*P* < 0.05) were used for multiple comparisons. The related primer was listed in Table S3. The 10 mutated *CTF1* promoter sequences were listed in Table S4.

### Phylogenetic and selection analysis of *CTF1*

Geographic distributions were visualised using the ggplot2 software package (https://learn.microsoft.com/en‐us/azure/databricks/visualizations/ggplot2). A panel of 307 rice from RDP2 (BioProject accession PRJNA985385), 36 *O. nivara* and 129 *O. rufipogon* accessions (Jing *et al*., 2023) was used to construct a minimum spanning tree for *CTF1*. We further extracted the target sequences using bcftools view function (Danecek *et al*., 2021). The DNA sequences were used to analyse the 1.2 kb region containing *CTF1*(1 k promoter and CDS).

Arlequin version 3.5 (Excoffier and Lischer, 2010) was used to calculate the minimum spanning tree among the defined haplotypes. Arlequin's distance matrix output associated with 13 polymorphism sites was used in geneHapR (https://github.com/ZhangRenL/geneHapR; Zhang *et al*., 2023) to draw a minimum spanning tree.

Nucleotide diversity, FST and Tajima's *D* were calculated by vcftools (v0.1.16) (https://vcftools.sourceforge.net; Danecek *et al*., 2011) with a sliding window of 5 kb and a step of 500 bp. The averaged nucleotide diversity was assessed for each subpopulation across three regions: a 20 kb upstream region, the 1.2 kb region containing *CTF1*, and a 20 kb downstream region. This analysis aimed to determine if different flanking region lengths affect nucleotide diversity detection. The examined groups included *indica*, *aus*, *japonica*, *O. nivara*, *and O. rufipogon* accessions.

We further conducted an infiltration analysis using Dsuite (Malinsky *et al*., 2021) based on a four‐taxon D statistic to identify genomic segments infiltration between *indica* and *japonica* and to clarify the transmission process of *CTF1*. *O. nivara* and *O. rufipogon* were used as outgroups to infer the ancestral and derived SNP allelic states in the *japonica* (P3), *aus* (P1), and *indica* (P2) populations with a sliding window of 1 kb and a step of 500 bp.

## Authors' contributions

JF D and TF Y performed most of the experiments and data analysis. SH Z, HF H, J W, RS L, J W, JS C, L Z, YM M, WH L, S N participated in material development, sample preparation and data analysis. SK W, GQ Z, B L and JL Z revised the manuscript. TF Y designed the experiments and drafted the manuscript. All authors read and approved the final manuscript.

## Funding

This study was partially supported by the National Natural Science Foundation of China (32072047), Guangdong Basic and Applied Basic Research Foundation (2020A1515011051, 2022A1515012135, 2022A1515012328), Seed industry revitalisation project of special fund for rural revitalisation strategy in Guangdong Province (2022NPY00005), Guangdong Key Laboratory of New Technology in Rice Breeding (2023B1212060042), Elite Rice Plan of Rice Research Institute, Guangdong Academy of Agricultural Sciences (2023YG01).

## Competing interests

The authors declare that they have no competing interests.

## Supporting information


**Figure S1** The development of materials.

**Figure S2** Setting temperature for cold treatment at the flowering stage.

**Figure S3** CSSL W12‐25 with stable cold tolerance phenotype.

**Figure S4** The intervals of *qCTF3* and *qCTF6*.

**Figure S5** The agronomic traits of HJX74 and S20‐9‐18.

**Figure S6** Comparison of sequence differences between HJX74 and S20‐9‐18 in CDS regions for three genes.

**Figure S7** There was no significant difference in the relative seed setting rates between HJX74 and *LOC_Os06g04240* (a and c) or *LOC_Os06g04200* (b and d) complementary lines after cold stress.

**Figure S8** The relative expression level of over expression lines (a) and the mutation type of knock out lines (b) of *CTF1*.

**Figure S9** The information of haplotype 6 to 11 of *CTF1* in wild rice.

**Figure S10** The infiltration of *CTF1* between *indica* and *japonica* subpopulations determined by using four‐taxon D statistic tests.

**Figure S11** The fixation index of genomic regions harbouring *CTF1*.

**Figure S12** *CTF1* reprograms global gene expression.

**Table S1** The four cold tolerance SSSLs used for haplotype analysis of *CTF1*.

**Table S2** The 307 accessions from RDP2 and 160 wild rice used for haplotype analysis of *CTF1*.

**Table S3** The primer sequences used in this study.

**Table S4** The 10 mutated *CTF1* promoter sequences based on S20‐9‐18 DNA sequences.

**Table S5** Average nucleotide diversity of *CTF1* and 20 kb flanking region.

**Table S6** Neutrality tests of *CTF1*.

## Data Availability

The data that supports the findings of this study are available in the Supplementary Material of this article.
