# Supplementary figures and images for: Natural variation in CTF1 conferring cold tolerance at the flowering stage in rice
Source: Plant Biotechnol J. 2025 Jan 29;23(5):1491–506. doi: 10.1111/pbi.14600 (PMC12018822; doi:10.1111/pbi.14600)

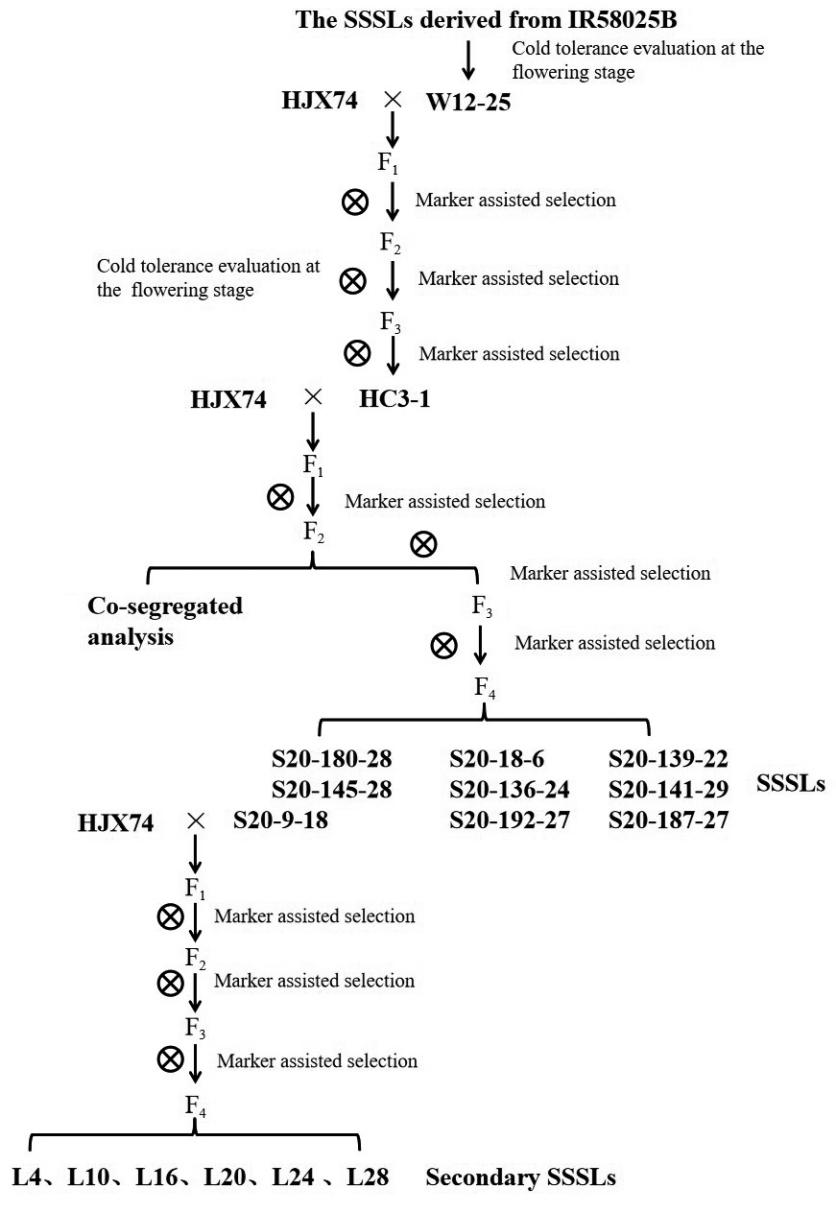


Fig.S1

Supplement: Supplementary file 1 — Figure S1 The development of materials. [file PBI-23-1491-s016.docx]

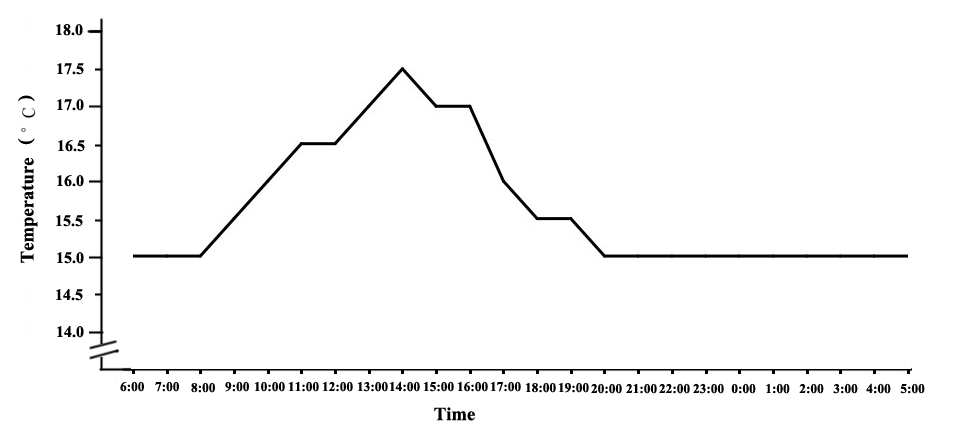


Fig.S2

Supplement: Supplementary file 2 — Figure S2 Setting temperature for cold treatment at the flowering stage. [file PBI-23-1491-s001.docx]

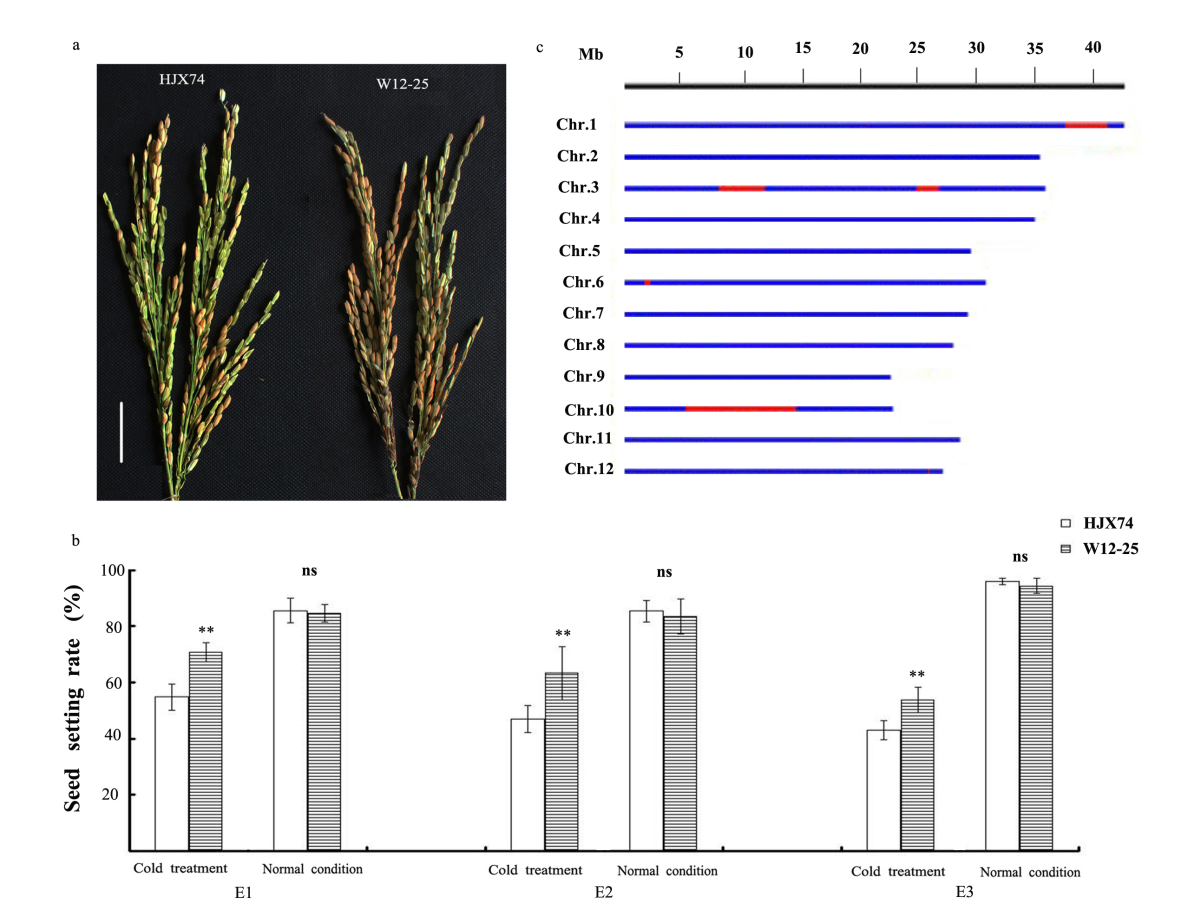


Fig.S3

Supplement: Supplementary file 3 — Figure S3 CSSL W12‐25 with stable cold tolerance phenotype. [file PBI-23-1491-s011.docx]

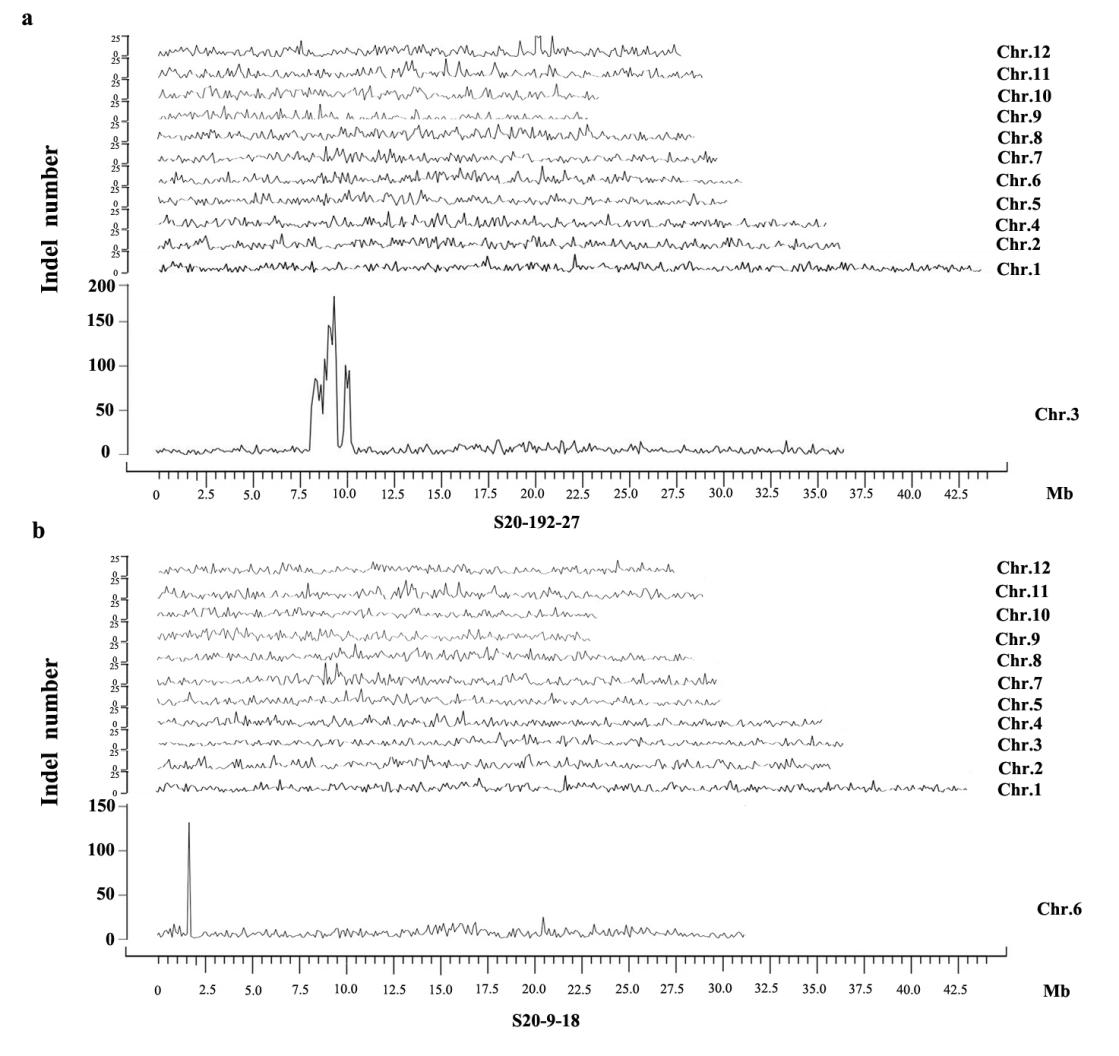


Fig.S4

Supplement: Supplementary file 4 — Figure S4 The intervals of qCTF3 and qCTF6. [file PBI-23-1491-s008.docx]

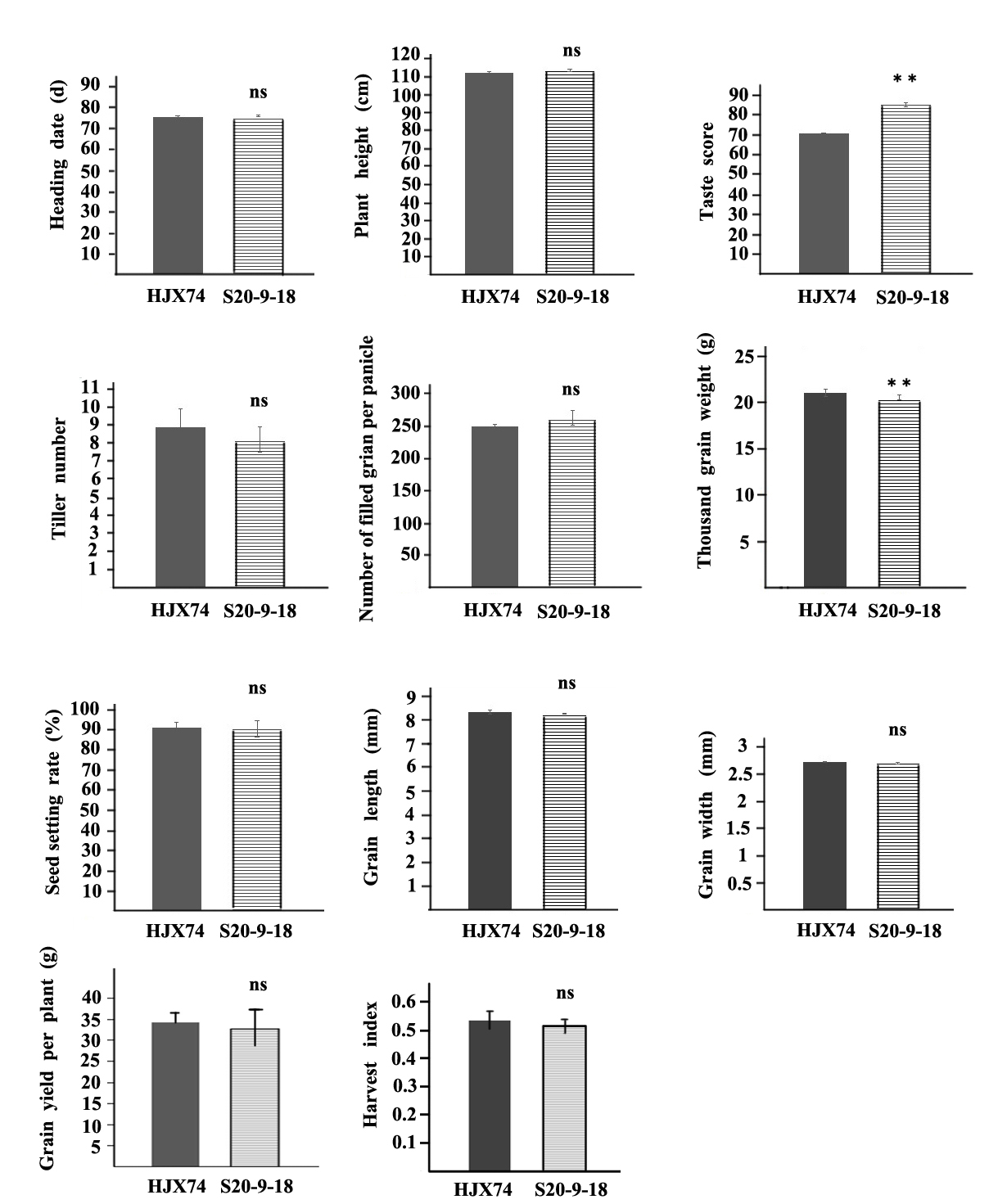


Fig.S5

Supplement: Supplementary file 5 — Figure S5 The agronomic traits of HJX74 and S20‐9‐18. [file PBI-23-1491-s014.docx]

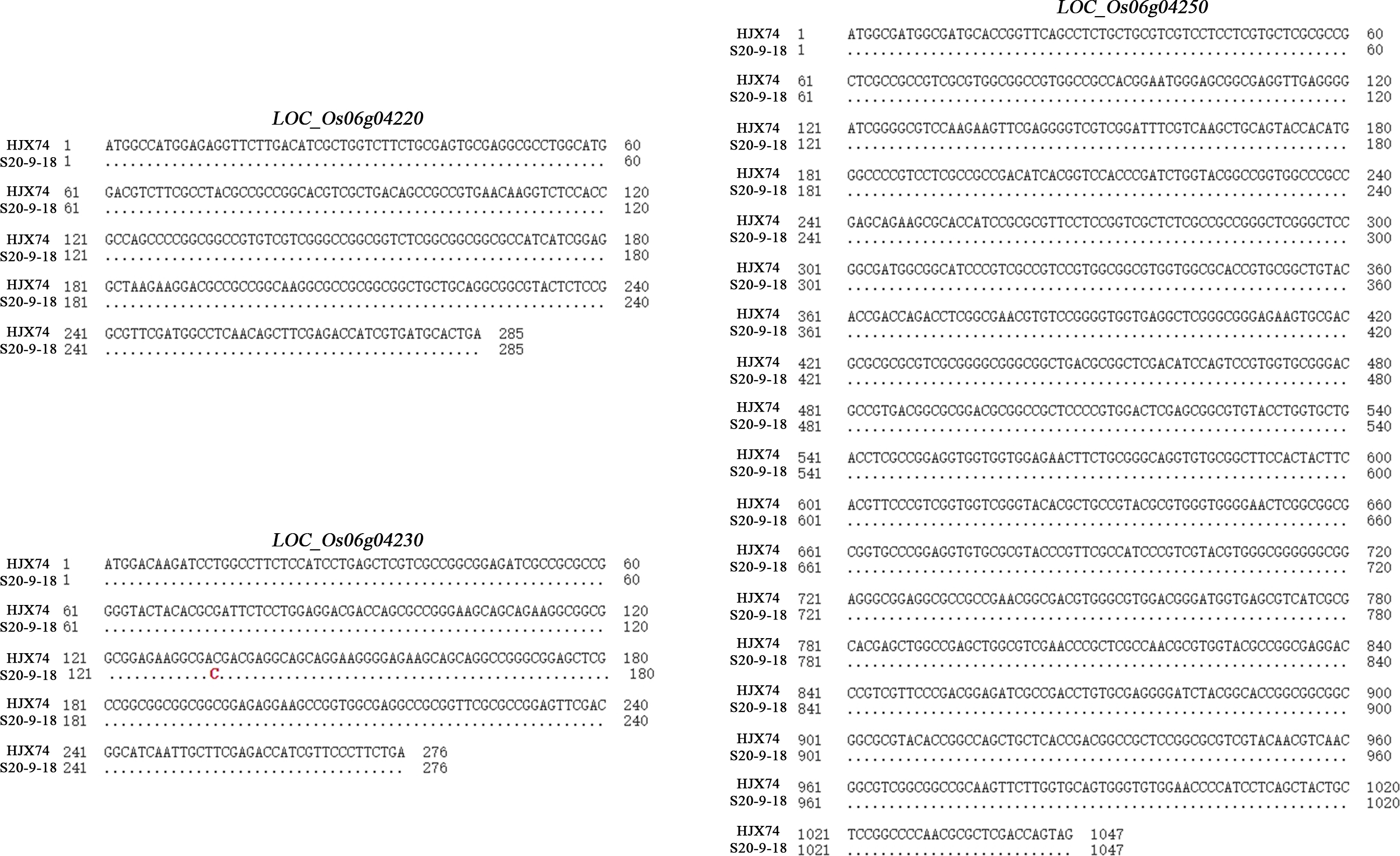


Fig.S6

Supplement: Supplementary file 6 — Figure S6 Comparison of sequence differences between HJX74 and S20‐9‐18 in CDS regions for three genes. [file PBI-23-1491-s015.docx]

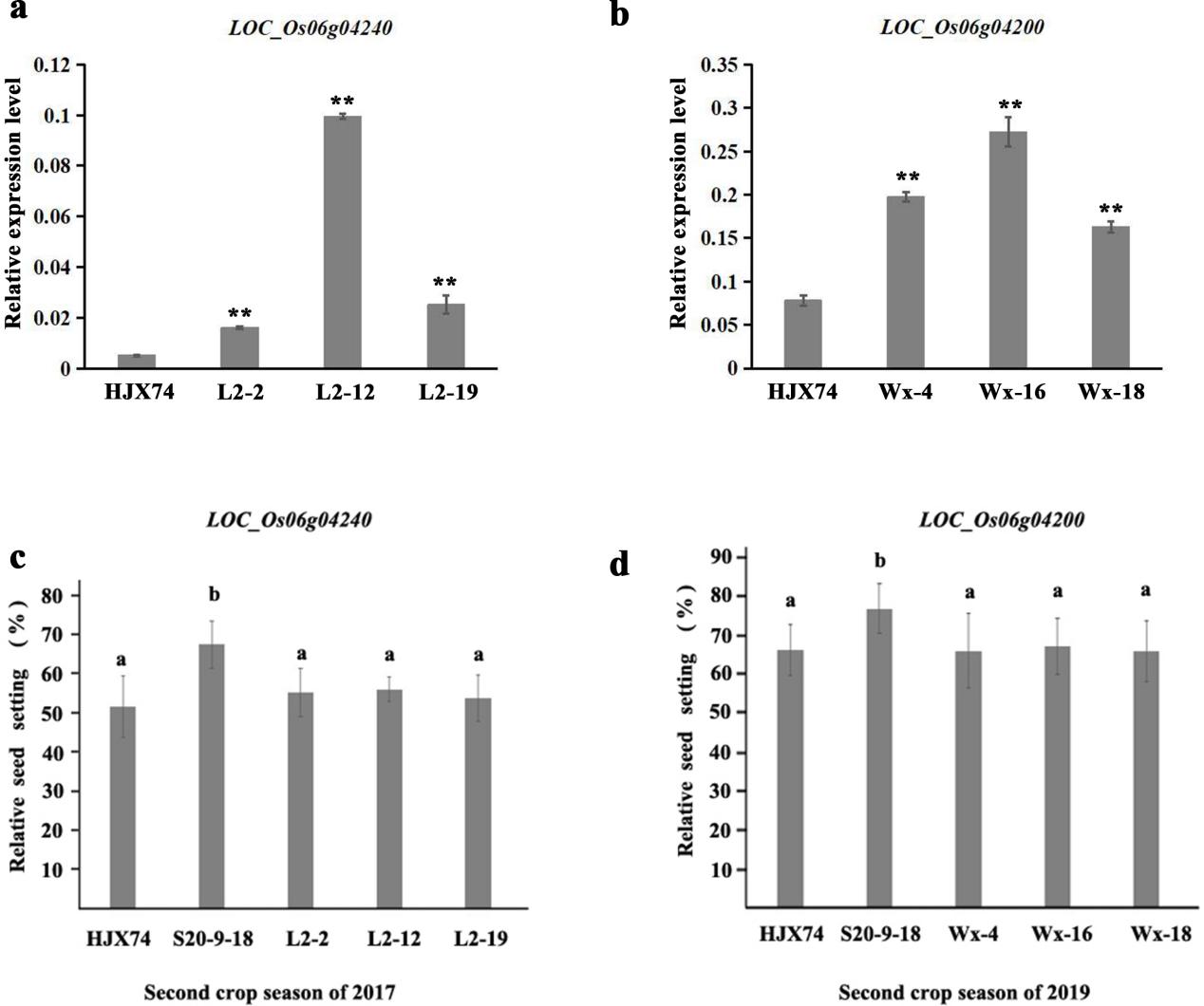


Fig.S7

Supplement: Supplementary file 7 — Figure S7 There was no significant difference in the relative seed setting rates between HJX74 and LOC_Os06g04240 (a and c) or LOC_Os06g04200 (b and d) complementary lines after cold stress. [file PBI-23-1491-s013.docx]

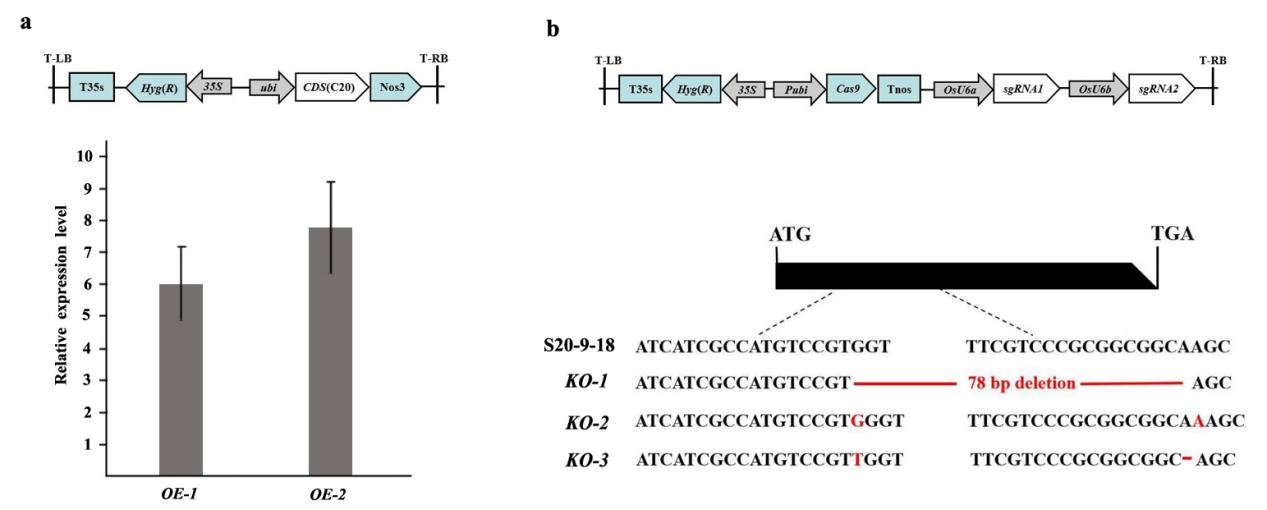


Fig.S8

Supplement: Supplementary file 8 — Figure S8 The relative expression level of over expression lines (a) and the mutation type of knock out lines (b) of CTF1. [file PBI-23-1491-s006.docx]

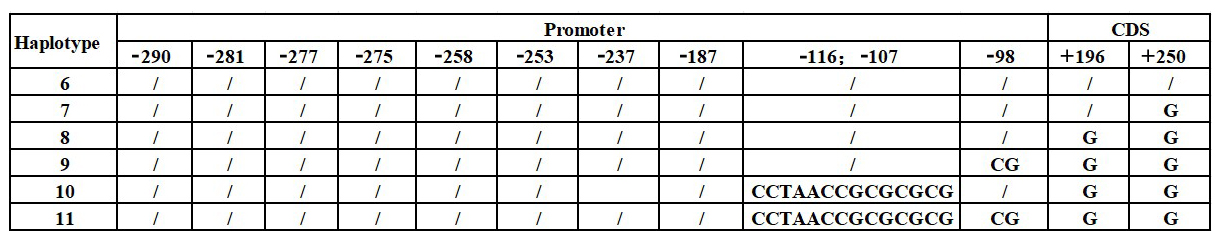


Fig.S9

Supplement: Supplementary file 9 — Figure S9 The information of haplotype 6 to 11 of CTF1 in wild rice. [file PBI-23-1491-s005.docx]

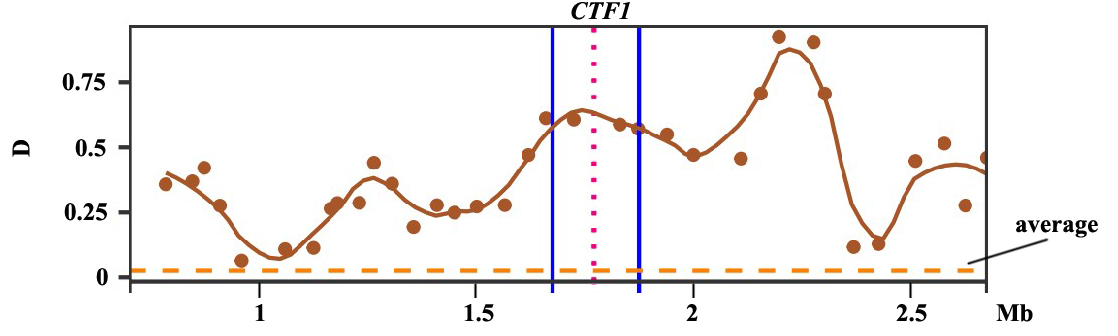


Fig.S10

Supplement: Supplementary file 10 — Figure S10 The infiltration of CTF1 between indica and japonica subpopulations determined by using four‐taxon D statistic tests. [file PBI-23-1491-s009.docx]

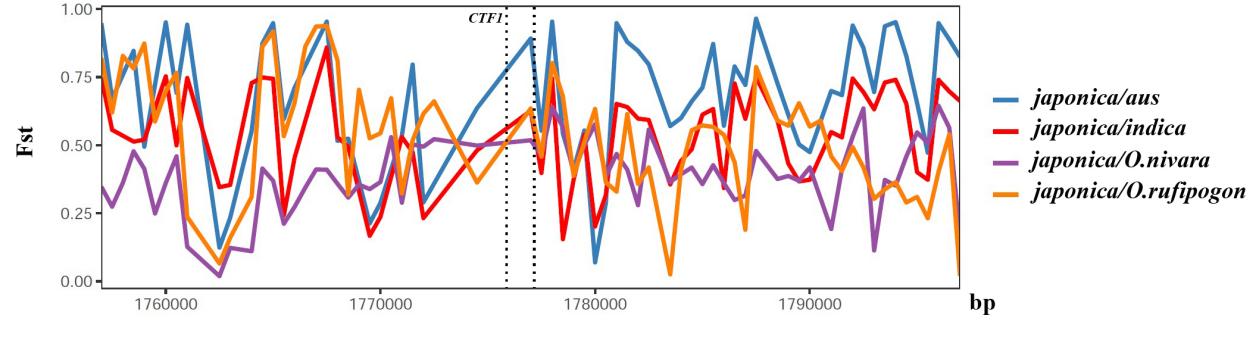


Fig.S11

Supplement: Supplementary file 11 — Figure S11 The fixation index of genomic regions harbouring CTF1. [file PBI-23-1491-s017.docx]

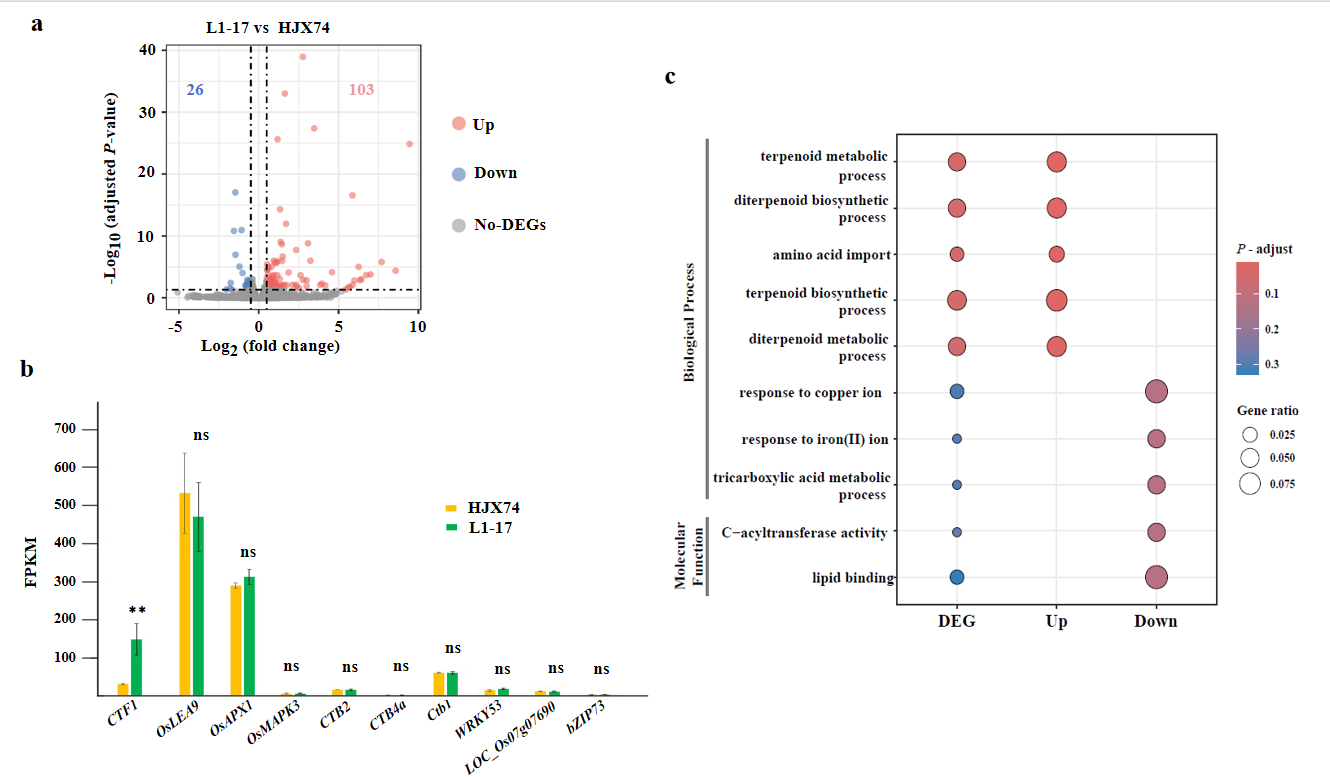


Fig.S12

Supplement: Supplementary file 12 — Figure S12 CTF1 reprograms global gene expression. [file PBI-23-1491-s004.docx]
