# Supplementary material for: Natural variation in CTF1 conferring cold tolerance at the flowering stage in rice
Source: Plant Biotechnol J. 2025 Jan 29;23(5):1491–506. doi: 10.1111/pbi.14600 (PMC12018822; doi:10.1111/pbi.14600)
Supplement: Supplementary file 13 — Table S1 The four cold tolerance SSSLs used for haplotype analysis of CTF1. [file PBI-23-1491-s007.docx]

Table S1 The four cold tolerance SSSLs used for haplotype analysis of *CTF1*

| Code | SSSL | Substitution segment | Donor |
| --- | --- | --- | --- |
| YH6 | W15-5-7-34 | Short arm--RM508-RM170-RM190-RM587-RM510--RM225 | American jasmine |
| YH7 | W17-10-7-5-12 | Short arm--RM133-RM589-RM190--RM587 | Ganxiangnuo |
| YH11 | W27-14-6-20 | RM589--RM190-RM204-RM225-RM253--RM402 | IAPAR9 |
| YH13 | W31-41-61-03-11-03-01 | PSM387--RM170-RM190-RM587-RM204-RM225-RM217-RM314--RM111 | IR66897B |
