# Supplementary material for: Natural variation in CTF1 conferring cold tolerance at the flowering stage in rice
Source: Plant Biotechnol J. 2025 Jan 29;23(5):1491–506. doi: 10.1111/pbi.14600 (PMC12018822; doi:10.1111/pbi.14600)
Supplement: Supplementary file 14 — Table S2 The 307 accessions from RDP2 and 160 wild rice used for haplotype analysis of CTF1. [file PBI-23-1491-s003.docx]

**Table S2** The 307 accessions from RDP2 and 160 wild rice used for haplotype analysis of *CTF1*

| In-house identify number | IRGC number | Accession name | Subpopulation | Country of origin | Haplotype  of *CTF1* |
| --- | --- | --- | --- | --- | --- |
| accessions from RDP2 |  |  |  |  |  |
| 6 | 117429 | AUS JOTA::IRGC 66767-1 | *aus* | Bangladesh | Hap1 |
| 7 | 117434 | BARAN BORO::IRGC 27509-1 | *aus* | Bangladesh | Hap1 |
| 30 | 117470 | FANDRAPOTSY::IRGC 10984-1 | *indica* | Madagascar | Hap4 |
| 37 | 117483 | HAIFUGOYA::IRGC 17054-1 | *japonica* | Taiwan,China | Hap5 |
| 39 | 117492 | INDANE::IRGC 33130-1 | *japonica* | Myanmar | Hap5 |
| 46 | 117502 | JHONA 26::IRGC 27967-1 | *aus* | Pakistan | Hap3 |
| 50 | 117513 | KHAO DAM::IRGC 23385-1 | *japonica* | Lao People's Democratic Republic | Hap5 |
| 56 | 117525 | MADAEL::IRGC 7722-1 | *indica* | Sri Lanka | Hap4 |
| 58 | 117528 | MALAGKIT PIRURUTONG::IRGC 8182-1 | *japonica* | Philippines | Hap5 |
| 62 | 117533 | MTU 9::IRGC 7919-1 | *indica* | India | Hap2 |
| 64 | 117539 | NHTA 5::IRGC 186-1 | *japonica* | India | Hap5 |
| 66 | 117545 | PACHEHAI PERUMAL::IRGC 8188-1 | *indica* | India | Hap4 |
| 78 | 117565 | RATHAL::IRGC 31524-1 | *japonica* | Sri Lanka | Hap5 |
| 79 | 117566 | RATHAL::IRGC 31525-1 | *japonica* | Sri Lanka | Hap5 |
| 82 | 117571 | RT 1031-69::IRGC 15092-1 | *japonica* | The Democratic Republic of the Congo | Hap5 |
| 86 | 117587 | TD 25::IRGC 9146-1 | *indica* | Thailand | Hap5 |
| 87 | 117588 | TEPI BORO::IRGC 27519-1 | *aus* | Bangladesh | Hap3 |
| 91 | 117598 | VANDANA::IRGC 117398-1 | *indica* | India | Hap1 |
| 414 | 120849 | AUS 196::IRGC 29016-1 | *aus* | Bangladesh | Hap2 |
| 423 | 120858 | 29 A 2::IRGC 28364-1 | *aus* | Pakistan | Hap3 |
| 436 | 120872 | ARC 15340::IRGC 42019-1 | *aus* | India | Hap3 |
| 440 | 120877 | AUS 55::IRGC 28918-1 | *aus* | Bangladesh | Hap1 |
| 441 | 120878 | B 6144 F-MR-6::IRGC 117313-1 | *indica* | Indonesia | Hap1 |
| 442 | 120879 | BADA DHAN::IRGC 26540-1 | *indica* | Bangladesh | Hap1 |
| 447 | 120886 | BANS 4::IRGC 74728-1 | *aus* | India | Hap1 |
| 452 | 120891 | BENAMURI::IRGC 25840-1 | *aus* | Bangladesh | Hap1 |
| 454 | 120893 | BG 34-11::IRGC 15782-1 | *indica* | Sri Lanka | Hap1 |
| 456 | 120895 | BIRAIN 360::IRGC 6550-1 | *indica* | Bangladesh | Hap2 |
| 461 | 120903 | BR IRGA 409::IRGC 116960-1 | *indica* | Brazil | Hap1 |
| 462 | 120904 | BYAT KYAR::IRGC 33004-1 | *indica* | Myanmar | Hap1 |
| 463 | 120905 | CAMPONI SML::IRGC 50640-1 | *indica* | Surinam | Hap5 |
| 467 | 120909 | CHI TOU HUANG 1::IRGC 51280-1 | *indica* | China | Hap4 |
| 468 | 120910 | CHIADI NAKI::IRGC 52101-1 | *aus* | India | Hap1 |
| 469 | 120911 | CHIKON SHONI::IRGC 64771-1 | *aus* | Bangladesh | Hap3 |
| 472 | 120914 | CHUA DAU::IRGC 4785-1 | *indica* | China | Hap4 |
| 476 | 120918 | CR 5272::IRGC 116971-1 | *indica* | Costa Rica | Hap3 |
| 477 | 120919 | CR 8334::IRGC 116972-1 | *indica* | Costa Rica | Hap1 |
| 483 | 120929 | DENG DENG QI::IRGC 72671-1 | *indica* | China | Hap1 |

| 484 | 120930 | DEVAREDDIRI::IRGC 40788-1 | *indica* | Sri Lanka | Hap4 |
| --- | --- | --- | --- | --- | --- |
| 489 | 120935 | DHARIA::IRGC 64773-1 | *aus* | Bangladesh | Hap3 |
| 491 | 120939 | DJOGOLON DJOGOLON::IRGC 75577-1 | *indica* | Burkina Faso | Hap1 |
| 493 | 120946 | E 5168::IRGC 68021-1 | *indica* | China | Hap1 |
| 494 | 120947 | E ZI 124::IRGC 70215-1 | *indica* | China | Hap2 |
| 500 | 120953 | FULKATI::IRGC 66783-1 | *aus* | Bangladesh | Hap1 |
| 501 | 120954 | GADRA::IRGC 73098-1 | *aus* | Pakistan | Hap3 |
| 504 | 120958 | GODA HEENATI::IRGC 31393-1 | *indica* | Sri Lanka | Hap4 |
| 505 | 120959 | GOPAL::IRGC 61953-1 | *indica* | Nepal | Hap1 |
| 507 | 120961 | GUL MURALI::IRGC 66792-1 | *aus* | Bangladesh | Hap1 |
| 510 | 120964 | HAO HOM::IRGC 12931-1 | *indica* | Laos | Hap5 |
| 516 | 120970 | HONG MI DONG MAO ZHAN::IRGC 68078-1 | *indica* | China | Hap1 |
| 517 | 120971 | HONG ZUI ER::IRGC 72706-1 | *indica* | China | Hap1 |
| 519 | 120973 | HUA KE ZAO::IRGC 76598-1 | *indica* | China | Hap1 |
| 522 | 120977 | ICTA POLOCHIC::IRGC 116997-1 | *indica* | Guatemala | Hap1 |
| 523 | 120978 | IKRA::IRGC 77250-1 | *aus* | Bangladesh | Hap1 |
| 525 | 120981 | IR 31917-45-3-2::IRGC 78132-1 | *indica* | Philippines | Hap2 |
| 527 | 120983 | IR 43::IRGC 117005-1 | *indica* | Philippines | Hap5 |
| 530 | 120986 | IR 74371-3-1-1::IRGC 117373-1 | *indica* | Philippines | Hap1 |
| 532 | 120988 | IR 77384-12-35-3-12-1-B::IRGC 117299-1 | *indica* | Philippines | Hap5 |
| 536 | 120995 | JAMBALI BUSSA::IRGC 73102-1 | *aus* | Pakistan | Hap3 |
| 539 | 120999 | JHUM BEGUNBICHI::IRGC 25867-1 | *aus* | Bangladesh | Hap1 |
| 548 | 121009 | KANGRO::IRGC 73105-1 | *aus* | Pakistan | Hap3 |
| 551 | 121013 | KASAPUR::IRGC 74751-1 | *aus* | India | Hap3 |
| 553 | 121015 | KATI::IRGC 67838-1 | *indica* | Bhutan | Hap2 |
| 554 | 121016 | KAUKHMWE::IRGC 33174-1 | *indica* | Myanmar | Hap5 |
| 555 | 121017 | KELE BARDHAN::IRGC 34983-1 | *aus* | India | Hap1 |
| 556 | 121018 | KELEE::IRGC 66807-1 | *aus* | Bangladesh | Hap1 |
| 557 | 121019 | KHAO DAW TAI::IRGC 24108-1 | *indica* | Thailand | Hap5 |
| 558 | 121020 | KHAO PON::IRGC 48114-1 | *indica* | Thailand | Hap5 |
| 561 | 121023 | KI-DANG PUTI::IRGC 44513-1 | *indica* | Philippines | Hap1 |
| 562 | 121024 | KIRIMURUNGA::IRGC 15585-1 | *indica* | Sri Lanka | Hap3 |
| 564 | 121026 | KOYRA::IRGC 77267-1 | *aus* | Bangladesh | Hap3 |
| 565 | 121027 | KUAN CHIN::IRGC 5589-1 | *indica* | Taiwan,China | Hap1 |
| 571 | 121034 | LAL BAGDAR::IRGC 77272-1 | *indica* | Bangladesh | Hap4 |
| 572 | 121035 | LALBAJAM::IRGC 49227-1 | *indica* | Bangladesh | Hap1 |
| 578 | 121041 | LOKU SAMBA::IRGC 31462-1 | *indica* | Sri Lanka | Hap2 |
| 581 | 121044 | MA GU ZI HE::IRGC 68212-1 | *indica* | China | Hap4 |
| 583 | 121046 | MADHABSAIL 741::IRGC 29374-1 | *aus* | Bangladesh | Hap1 |
| 585 | 121049 | MAKALIOKA::IRGC 77864-1 | *indica* | Madagascar | Hap4 |
| 588 | 121052 | MENAKELY::IRGC 69963-1 | *indica* | Madagascar | Hap4 |
| 589 | 121053 | MG 2::IRGC 79837-1 | *indica* | Brazil | Hap5 |
| 593 | 121057 | MOSHIA BHADOI::IRGC 66817-1 | *aus* | Bangladesh | Hap1 |

| 594 | 121058 | MOSHUR::IRGC 64789-1 | *aus* | Bangladesh | Hap1 |
| --- | --- | --- | --- | --- | --- |
| 595 | 121059 | MOTTA SAMBA::IRGC 36489-1 | *indica* | Sri Lanka | Hap4 |
| 598 | 121062 | NAKABAWA::IRGC 70676-1 | *indica* | Kenya | Hap5 |
| 599 | 121063 | NAN TE 113::IRGC 70345-1 | *indica* | China | Hap5 |
| 606 | 121070 | NORUNKAN::IRGC 8934-1 | *indica* | Sri Lanka | Hap1 |
| 607 | 121071 | NP 125::IRGC 32559-1 | *aus* | India | Hap3 |
| 611 | 121076 | NSICRC 106::IRGC 117370-1 | *indica* | Philippines | Hap1 |
| 612 | 121077 | PANAKALI::IRGC 47399-1 | *indica* | Sri Lanka | Hap4 |
| 613 | 121078 | PANKHIRAJ::IRGC 25911-1 | *aus* | Bangladesh | Hap3 |
| 615 | 121080 | PAWHTUN::IRGC 33562-1 | *indica* | Myanmar | Hap1 |
| 616 | 121081 | PERLA::IRGC 117021-1 | *indica* | Cuba | Hap5 |
| 620 | 121085 | PICONEGRO::IRGC 117022-1 | *indica* | Ecuador | Hap1 |
| 625 | 121091 | PURBIA (KALANSAR)::IRGC 59189-1 | *indica* | Nepal | Hap1 |
| 630 | 121096 | RAKHOIL::IRGC 64793-1 | *aus* | Bangladesh | Hap1 |
| 632 | 121098 | RATHKANDIRAM::IRGC 36507-1 | *indica* | Sri Lanka | Hap1 |
| 633 | 121099 | RAY JAZAYKAYZ::IRGC 62181-1 | *indica* | Bhutan | Hap1 |
| 640 | 121107 | SADA SOLAY::IRGC 73118-1 | *aus* | Pakistan | Hap1 |
| 643 | 121110 | SAN DU BAI MI HONG GU::IRGC 59849-1 | *indica* | China | Hap1 |
| 645 | 121112 | SANTHI SUFAID 207::IRGC 28212-1 | *aus* | Pakistan | Hap3 |
| 651 | 121120 | SIMUL KHURI::IRGC 35154-1 | *aus* | India | Hap3 |
| 653 | 121122 | SOKOU MALSIRA::IRGC 77301-1 | *indica* | Bangladesh | Hap1 |
| 655 | 121124 | SOMIMADAMO::IRGC 69044-1 | *indica* | Madagascar | Hap4 |
| 656 | 121125 | SPR 87032-2-1-1-4::IRGC 117355-1 | *indica* | Thailand | Hap5 |
| 667 | 121137 | TI KU::IRGC 1224-1 | *indica* | China | Hap4 |
| 673 | 121145 | TUNG CH'IU AI::IRGC 34265-1 | *aus* | China | Hap3 |
| 679 | 121151 | VARY VATO MENAHODITRA::IRGC 69111-1 | *indica* | Madagascar | Hap4 |
| 680 | 121152 | VELLAI SEENETTI::IRGC 15516-1 | *indica* | Sri Lanka | Hap4 |
| 681 | 121153 | WANNI DAHANALA::IRGC 15721-1 | *indica* | Sri Lanka | Hap4 |
| 683 | 121156 | XI GU HONG::IRGC 74226-1 | *indica* | China | Hap1 |
| 684 | 121157 | XI NUO ZAO::IRGC 68279-1 | *indica* | China | Hap1 |
| 685 | 121158 | YAKADA::IRGC 51096-1 | *indica* | Sri Lanka | Hap1 |
| 686 | 121159 | YE TI ZHAN::IRGC 68296-1 | *indica* | China | Hap1 |
| 687 | 121161 | ZALCHA::IRGC 62190-1 | *indica* | Bhutan | Hap1 |
| 688 | 121162 | ZAO SHAO ZHAN::IRGC 68318-1 | *indica* | China | Hap1 |
| 689 | 121163 | ZAO SHOU 691-11::IRGC 70447-1 | *indica* | China | Hap1 |
| 690 | 121164 | ZI GAN - GU::IRGC 70468-1 | *indica* | China | Hap1 |
| 694 | 121170 | KAUKYAGYI::IRGC 33208-1 | *indica* | Myanmar | Hap5 |
| 700 | 121176 | XI GU ZAO::IRGC 72360-1 | *indica* | China | Hap1 |
| 702 | 121182 | 1-52-6::IRGC 39111-1 | *japonica* | Brazil | Hap5 |
| 704 | 121184 | AI QING 99::IRGC 63544-1 | *indica* | China | Hap1 |
| 706 | 121186 | ARC 11559::IRGC 21477-1 | *aus* | India | Hap1 |
| 711 | 121191 | AZMIL 85::IRGC 289-1 | *japonica* | Philippines | Hap5 |
| 716 | 121196 | DUANG OJOY::IRGC 3832-1 | *japonica* | Philippines | Hap5 |

| 717 | 121197 | DULAR::IRGC 32561-1 | *aus* | India | Hap1 |
| --- | --- | --- | --- | --- | --- |
| 725 | 121205 | IC 27525::IRGC 53989-1 | *aus* | India | Hap1 |
| 726 | 121206 | JHUM SONALICHIKON::IRGC 25874-1 | *aus* | Bangladesh | Hap1 |
| 730 | 121210 | MERCURY::IRGC 77141-1 | *japonica* | United States of America | Hap5 |
| 731 | 121211 | MIKHUDEB::IRGC 25892-1 | *japonica* | Bangladesh | Hap5 |
| 733 | 121213 | MO WAE DO::IRGC 2356-1 | *japonica* | Republic of Korea | Hap5 |
| 740 | 121220 | SOKANA::IRGC 52763-1 | *aus* | India | Hap3 |
| 745 | 121225 | THAPACHINIYA::IRGC 16234-1 | *indica* | Nepal | Hap1 |
| 752 | 121232 | DHOLI BORO::IRGC 27513-2 | *aus* | Bangladesh | Hap2 |
| 755 | 121235 | KHAO DAWK MALI 105::IRGC 27748-2 | *indica* | Thailand | Hap5 |
| 761 | 121241 | 62-667::IRGC 15147-1 | *japonica* | Ivory Coast | Hap5 |
| 762 | 121242 | 7507-137::IRGC 40081-1 | *japonica* | Japan | Hap5 |
| 787 | 121267 | ARGO::IRGC 82418-1 | *japonica* | Italy | Hap5 |
| 794 | 121274 | BAGA- ADONGKO::IRGC 71501-1 | *japonica* | Malaysia | Hap5 |
| 798 | 121278 | BALAYAN::IRGC 71505-1 | *japonica* | Malaysia | Hap5 |
| 801 | 121281 | BANAT 2270::IRGC 65648-1 | *japonica* | Romania | Hap5 |
| 806 | 121286 | BEN DI HONG KE NUO::IRGC 70065-1 | *japonica* | China | Hap5 |
| 810 | 121290 | BOSSA::IRGC 57781-1 | *japonica* | Guinea | Hap5 |
| 812 | 121292 | BOTPA BARA::IRGC 62162-1 | *japonica* | Bhutan | Hap5 |
| 813 | 121293 | BOTRA FOTSY::IRGC 77840-1 | *japonica* | Madagascar | Hap5 |
| 821 | 121301 | C 8434::IRGC 13496-1 | *japonica* | Papua New Guinea | Hap5 |
| 822 | 121302 | CAIAPO::IRGC 116962-1 | *japonica* | Brazil | Hap5 |
| 823 | 121303 | CALMOCHI 202::IRGC 57082-1 | *japonica* | United States of America | Hap5 |
| 829 | 121309 | CHANGYOUNG 7::IRGC 82284-1 | *japonica* | Republic of Korea | Hap5 |
| 831 | 121311 | CHI- 2::IRGC 54656-1 | *indica* | India | Hap4 |
| 837 | 121317 | CNAR 2888-B-47::IRGC 117325-1 | *japonica* | Brazil | Hap5 |
| 844 | 121324 | DEHULA::IRGC 74737-1 | *aus* | India | Hap3 |
| 846 | 121326 | DOBONGBYEO (SUWEON 223)::IRGC 58315-1 | *japonica* | Republic of Korea | Hap5 |
| 847 | 121327 | DOONGARA::IRGC 78392-1 | *japonica* | australia | Hap5 |
| 851 | 121331 | DULAR::IRGC 636-1 | *aus* | India | Hap1 |
| 852 | 121332 | DUMSIKALAM::IRGC 58968-1 | *indica* | Nepal | Hap1 |
| 854 | 121334 | ELVO::IRGC 82422-1 | *japonica* | Italy | Hap5 |
| 861 | 121341 | GBANTE::IRGC 16081-1 | *japonica* | Cote d'Ivoire | Hap5 |
| 871 | 121351 | HE JIANG 15::IRGC 61801-1 | *japonica* | China | Hap5 |
| 874 | 121354 | HOMURA 8::IRGC 5388-1 | *japonica* | Japan | Hap5 |
| 876 | 121356 | HUANG JING NUO::IRGC 80953-1 | *japonica* | China | Hap5 |
| 880 | 121360 | IAC 164::IRGC 55860-1 | *japonica* | Brazil | Hap5 |
| 883 | 121363 | INUWAY::IRGC 67437-1 | *japonica* | Philippines | Hap5 |
| 885 | 121365 | IRAT 170::IRGC 64850-1 | *japonica* | Cote d'Ivoire | Hap5 |
| 893 | 121373 | JI BO YA::IRGC 77446-1 | *japonica* | Congo | Hap5 |
| 896 | 121376 | JUMA::IRGC 66800-1 | *aus* | Bangladesh | Hap3 |
| 897 | 121377 | KAM MRA::IRGC 62172-1 | *japonica* | Bhutan | Hap5 |
| 902 | 121382 | KHADASIYA 3 (STRAW)::IRGC 54072-1 | *aus* | India | Hap3 |

| 903 | 121383 | Khang khouay::IRGC 29973-1 | *indica* | Laos | Hap5 |
| --- | --- | --- | --- | --- | --- |
| 907 | 121387 | KHAU THEP::IRGC 78341-1 | *indica* | Viet Nam | Hap1 |
| 908 | 121388 | Khie tom::IRGC 11887-1 | *indica* | Lao People's Democratic Republic | Hap5 |
| 912 | 121392 | KOMOL BHOG::IRGC 77266-1 | *indica* | Bangladesh | Hap4 |
| 916 | 121396 | KPOGON::IRGC 30467-1 | *japonica* | Liberia | Hap5 |
| 919 | 121399 | KULU::IRGC 11337-1 | *japonica* | australia | Hap5 |
| 922 | 121402 | KYRMYZY::IRGC 36164-1 | *japonica* | Uzbekistan | Hap5 |
| 924 | 121404 | LADY WRIGHT SELN (CI 12187)::IRGC 1761-1 | *japonica* | United States | Hap5 |
| 929 | 121409 | LEVANTE HOMEM::IRGC 50492-1 | *japonica* | Brazil | Hap5 |
| 932 | 121412 | LIGERITO (CORTO PUBESCENTE)::IRGC 19919-1 | *japonica* | Colombia | Hap1 |
| 940 | 121420 | MAHSURI::IRGC 10929-1 | *indica* | Malaysia | Hap1 |
| 941 | 121421 | MAI HSI TSAN::IRGC 51366-1 | *indica* | China | Hap1 |
| 942 | 121422 | MALACHAN::IRGC 54748-1 | *indica* | India | Hap4 |
| 949 | 121429 | MAWTAIK::IRGC 33359-1 | *indica* | Myanmar | Hap1 |
| 953 | 121433 | MIMIDIM::IRGC 25898-1 | *japonica* | Bangladesh | Hap5 |
| 981 | 121461 | ORIENTE 10::IRGC 55808-1 | *japonica* | Ecuador | Hap5 |
| 988 | 121468 | PECOS::IRGC 66758-1 | *japonica* | United States of America | Hap5 |
| 989 | 121469 | Peek::IRGC 11821-1 | *japonica* | Lao People's Democratic Republic | Hap5 |
| 1003 | 121483 | RHS 107-2-1-2TB-1JM::IRGC 117025-1 | *japonica* | Mexico | Hap5 |
| 1006 | 121486 | RIZZOTTO 264::IRGC 65727-1 | *japonica* | Italy | Hap5 |
| 1008 | 121488 | RXAR RGUE::IRGC 1943-1 | *japonica* | United States of America | Hap5 |
| 1015 | 121495 | SAREINA::IRGC 67757-1 | *aus* | India | Hap3 |
| 1025 | 121505 | SSAL BYEO 2::IRGC 19868-1 | *japonica* | Korea Rep | Hap5 |
| 1026 | 121506 | SUDU HEENATI::IRGC 15675-1 | *indica* | Sri Lanka | Hap5 |
| 1031 | 121511 | SUWEON 251::IRGC 46964-1 | *indica* | Korea Rep | Hap5 |
| 1037 | 121517 | TAI- IKU 512::IRGC 2993-1 | *japonica* | Taiwan,China | Hap3 |
| 1043 | 121523 | TOANG::IRGC 19144-1 | *japonica* | Indonesia | Hap5 |
| 1044 | 121524 | TOS 10483::IRGC 56723-1 | *japonica* | Guinea-Bissau | Hap5 |
| 1046 | 121526 | TOX 2104-2-1::IRGC 117257-1 | *japonica* | Nigeria | Hap5 |
| 1047 | 121527 | TSON HSU DAU::IRGC 4711-1 | *indica* | China | Hap4 |
| 1048 | 121528 | TUO TUO HUANG::IRGC 72851-1 | *indica* | China | Hap1 |
| 1054 | 121534 | VELA::IRGC 82437-1 | *japonica* | Italy | Hap5 |
| 1056 | 121536 | WAB 176-8-HB::IRGC 117358-1 | *japonica* | Cote d'Ivoire | Hap5 |
| 1058 | 121538 | WAB 99-16::IRGC 117360-1 | *japonica* | Cote d'Ivoire | Hap5 |
| 1061 | 121541 | WIR 1072::IRGC 57496-1 | *japonica* | Belgium | Hap5 |
| 1064 | 121544 | WIR 2521::IRGC 51658-1 | *japonica* | Ukraine | Hap5 |
| 1069 | 121549 | YAKUMO::IRGC 5320-1 | *japonica* | Japan | Hap5 |
| 1074 | 121554 | YU WEOL ZO::IRGC 19894-1 | *japonica* | Republic of Korea | Hap5 |
| 1075 | 121555 | ZHEN JIANG 2::IRGC 53439-1 | *indica* | China | Hap1 |
| 1079 | 121559 | ARC 11708::IRGC 21575-1 | *japonica* | India | Hap5 |
| 1087 | 121567 | KETAN MERAH::IRGC 24977-1 | *japonica* | Indonesia | Hap5 |
| 1089 | 121569 | L 201::IRGC 51099-1 | *japonica* | United States of America | Hap5 |
| 1096 | 121576 | NGAJA::IRGC 64917-1 | *indica* | Bhutan | Hap5 |

| 1098 | 121578 | OOURA::IRGC 501-1 | *japonica* | Japan | Hap5 |
| --- | --- | --- | --- | --- | --- |
| 1099 | 121579 | PRETO REGADO 142::IRGC 65724-1 | *japonica* | Morocco | Hap5 |
| 1100 | 121580 | RIENALDO BERZANO::IRGC 3230-1 | *japonica* | Turkey | Hap5 |
| 1114 | 121594 | ARC 10176::IRGC 20765-1 | *japonica* | India | Hap5 |
| 1117 | 121597 | ARC 14500::IRGC 42957-1 | *indica* | India | Hap2 |
| 1120 | 121600 | BENGIZA::IRGC 69845-1 | *japonica* | Madagascar | Hap5 |
| 1125 | 121605 | CHANDARHAT::IRGC 25845-1 | *aus* | Bangladesh | Hap1 |
| 1131 | 121611 | E KHA KEHA::IRGC 2764-1 | *indica* | Taiwan,China | Hap1 |
| 1135 | 121615 | IR 65192-4B-10-3::IRGC 117367-1 | *indica* | Philippines | Hap2 |
| 1137 | 121617 | IR 73678-20-1-B::IRGC 117292-1 | *indica* | Philippines | Hap1 |
| 1141 | 121621 | JOALBHANGA 499::IRGC 6560-1 | *indica* | Bangladesh | Hap5 |
| 1143 | 121623 | KAPUTU HOTA::IRGC 66516-1 | *indica* | Sri Lanka | Hap1 |
| 1148 | 121628 | MRT 195::IRGC 79799-1 | *indica* | Mauritania | Hap3 |
| 1165 | 121645 | TARA::IRGC 59244-1 | *indica* | Nepal | Hap5 |
| 1166 | 121646 | TOS 5790::IRGC 117256-1 | *japonica* | Nigeria | Hap5 |
| 1168 | 121648 | VARY MAINTY::IRGC 69910-1 | *japonica* | Madagascar | Hap5 |
| 1170 | 121650 | WU BAI LI::IRGC 70404-1 | *indica* | China | Hap1 |
| 1173 | 121653 | KHAO PAHK MAW | *aus* | Thailand | Hap3 |
| 1203 | 121683 | SR 26 B::IRGC 10798-1 | *indica* | Japan | Hap1 |
| 1214 | 121694 | PACHOLINHA::IRGC 50531-1 | *japonica* | Brazil | Hap5 |
| 1216 | 121696 | CURINCA::C1 | *japonica* | Brazil | Hap5 |
| 1219 | 121699 | MANDRIRAVINA 3512::GERVEX 8319-C1 | *japonica* | Madagascar | Hap5 |
| 1222 | 121702 | ADAIR::GERVEX 1640-C1 | *japonica* | United States of America | Hap5 |
| 1226 | 121706 | ARAGUAIA::IRTP 17399-C1 | *japonica* | Brazil | Hap5 |
| 1235 | 121715 | BENGALY VAKARINA::GERVEX 4750-C1 | *japonica* | Madagascar | Hap5 |
| 1237 | 121717 | BODOMANO::GERVEX 8343-C1 | *indica* | Madagascar | Hap1 |
| 1250 | 121730 | CIRAD 488::GERVEX 9172-C1 | *japonica* | Madagascar | Hap5 |
| 1251 | 121731 | COLINA::GERVEX 524-C1 | *japonica* | Spain | Hap5 |
| 1252 | 121732 | DELLROSE::GERVEX 1648-C1 | *japonica* | United States of America | Hap5 |
| 1254 | 121734 | DIXIEBELLE::GERVEX 1649-C1 | *japonica* | United States of America | Hap4 |
| 1257 | 121737 | FOHISOMOTRA::GERVEX 8329-C1 | *japonica* | Madagascar | Hap5 |
| 1261 | 121741 | GOGO LEMPAK::IRGC 43392-C1 | *japonica* | Indonesia | Hap5 |
| 1262 | 121742 | GUARANI::GERVEX 8506-C1 | *japonica* | Brazil | Hap5 |
| 1264 | 121744 | HD 1-4::C1 | *japonica* | France | Hap5 |
| 1267 | 121747 | ILANG-ILANG::GERVEX 509-C1 | *japonica* | Republic of Korea | Hap5 |
| 1268 | 121748 | IR 2006-P12-12-2::IRGC 32675-C1 | *indica* | Philippines | Hap1 |
| 1270 | 121750 | IR 50::C1 | *indica* | Philippines | Hap1 |
| 1272 | 121752 | IR 55419-04::C1 | *indica* | Philippines | Hap1 |
| 1273 | 121753 | IR 60::IRGC 63493-C1 | *indica* | Philippines | Hap1 |
| 1275 | 121755 | IR 63372-8::C1 | *japonica* | Philippines | Hap5 |
| 1276 | 121756 | IR 65261-09-1-B::C1 | *japonica* | Philippines | Hap1 |
| 1279 | 121759 | IR 71676-90-2-2::C1 | *indica* | Philippines | Hap5 |
| 1281 | 121761 | IRAT 109::GERVEX 4988-C1 | *japonica* | Cote d'Ivoire | Hap5 |

| 1283 | 121763 | IRAT 212::GERVEX 7698-C1 | *japonica* | Cote d'Ivoire | Hap5 |
| --- | --- | --- | --- | --- | --- |
| 1284 | 121764 | IRAT 216::GERVEX 7702-C1 | *japonica* | Ivory Coast | Hap5 |
| 1286 | 121766 | IRAT 2::GERVEX 606-C1 | *japonica* | Senegal | Hap5 |
| 1288 | 121768 | IRAT 364::GERVEX 8714-C1 | *japonica* | Nicaragua | Hap5 |
| 1291 | 121771 | JAMAJIGI::C1 | *indica* | Mali | Hap1 |
| 1292 | 121772 | JIMBRUK JOLOWORO::IRGC 43420-C1 | *japonica* | Indonesia | Hap5 |
| 1298 | 121778 | KITRANA 1890::GERVEX 5115-C1 | *indica* | Madagascar | Hap2 |
| 1301 | 121781 | KUROKA::IRGC 74556-C1 | *japonica* | Japan | Hap5 |
| 1302 | 121782 | KYEEMA::GERVEX 1656-C1 | *japonica* | australia | Hap5 |
| 1307 | 121787 | LUDAN::IRGC 64189-C1 | *japonica* | Philippines | Hap5 |
| 1310 | 121790 | M 204::GERVEX 1665-C1 | *japonica* | United States | Hap5 |
| 1313 | 121793 | MA-ELATRA 520::GERVEX 8445-C1 | *japonica* | Madagascar | Hap5 |
| 1320 | 121800 | OLCENENGO::GERVEX 41-C1 | *japonica* | Italy | Hap5 |
| 1321 | 121801 | ORYZICA SABANA 6::C1 | *japonica* | Colombia | Hap5 |
| 1323 | 121803 | PALAWAN::C1 | *japonica* | Philippines | Hap5 |
| 1325 | 121805 | PCT 4\SA\4\1>1076-2-4-1-5::C1 | *japonica* | Colombia | Hap5 |
| 1327 | 121807 | PEH PI NUO::IRGC 8266-C1 | *japonica* | China | Hap5 |
| 1329 | 121809 | PRIMAVERA::C1 | *japonica* | Brazil | Hap5 |
| 1332 | 121812 | ROJOMENA 1034::GERVEX 8412-C1 | *indica* | Madagascar | Hap4 |
| 1334 | 121814 | ROVUMA::GERVEX 1245-C1 | *japonica* | Portugal | Hap5 |
| 1339 | 121824 | SOURE::GERVEX 1294-C1 | *japonica* | Portugal | Hap5 |
| 1341 | 121826 | SUWEON 280::GERVEX 1306-C1 | *indica* | Republic of Korea | Hap5 |
| 1342 | 121827 | TELIMANI::C1 | *indica* | Mali | Hap2 |
| 1347 | 121832 | TSAKA::IRGC 64934-C1 | *indica* | Bhutan | Hap1 |
| 1348 | 121833 | TSIPALA 89::GERVEX 8385-C1 | *japonica* | Madagascar | Hap5 |
| 1353 | 121838 | VARY SOMOTRA SIHANAKA::GERVEX 8321-C1 | *japonica* | Madagascar | Hap5 |
| 1354 | 121839 | VARY VATO 154::GERVEX 5429-C1 | *indica* | Madagascar | Hap3 |
| 1360 | 121845 | WAB 706-3-4-K4-KB-1::C1 | *japonica* | Cote d'Ivoire | Hap5 |
| 1364 | 121849 | WAS 183-B-6-2-3::C1 | *indica* | Senegal | Hap5 |
| 1365 | 121850 | WAS 194-B-3-2-5::C1 | *indica* | Senegal | Hap2 |
| 1367 | 121852 | WAS 200-B-B-1-1-1::C1 | *indica* | Senegal | Hap1 |
| 1371 | 121856 | WAS 20-B-B-1-2-2::C1 | *indica* | Senegal | Hap2 |
| 1372 | 121857 | WAS 30-11-4-6-2-2-1::C1 | *indica* | Senegal | Hap2 |
| 1373 | 121858 | WAS 62-B-B-17-1-1-3::C1 | *indica* | Senegal | Hap2 |
| 1374 | 121859 | YANCAOUSSA::IRGC 16071-C1 | *japonica* | Cote d'Ivoire | Hap5 |
| 1377 | 121862 | ZENA::GERVEX 101-C1 | *japonica* | Italy | Hap5 |
| 1380 | 121865 | CODE NO 31281::IRGC 46891-1 | *indica* | India | Hap4 |
| 1388 | 121873 | BAKAW::IRGC 11169-1 | *japonica* | Philippines | Hap5 |
| 1390 | 121875 | BORUBI::IRGC25181-1 | *japonica* | Indonesia | Hap5 |
| 1394 | 121879 | IA CUBA 23::IRGC 116991-1 | *indica* | Cuba | Hap5 |
| 1395 | 121880 | IR 72132-AC 6-1::IRGC 117369-1 | *indica* | Philippines | Hap1 |
| 1400 | 121885 | MAHUDU KIRIYAL::IRGC 40844-1 | *indica* | Sri Lanka | Hap4 |
| 1403 | 121888 | PANAMA 1537::IRGC 117019-1 | *indica* | Panama | Hap1 |

| 1409 | 121894 | 11-049::IRGC 36400-1 | *indica* | Sri Lanka | Hap1 |
| --- | --- | --- | --- | --- | --- |
| 1416 | 121901 | BEKASAKA 158::IRGC 68361-1 | *indica* | Madagascar | Hap1 |
| 1420 | 121905 | CINA::IRGC 27116-1 | *japonica* | Indonesia | Hap5 |
| 1431 | 121916 | JUMA 58::IRGC 117010-1 | *indica* | Dominican Republic | Hap1 |
| 1434 | 121919 | KHAU MUONG PIENG::IRGC 78333-1 | *indica* | Viet Nam | Hap5 |
| 1435 | 121920 | KUMBIPHOU::IRGC 51990-1 | *indica* | India | Hap1 |
| 1438 | 121923 | NEP ME HOA BINH::IRGC 78366-1 | *japonica* | Viet Nam | Hap5 |
| 1442 | 121927 | PATO DE GALLINAZO Y 5371::IRGC 5766-1 | *japonica* | australia | Hap5 |
| 1445 | 121930 | P. TINGAGEW DAYKET QAY DAYON::IRGC 8046 | *japonica* | Philippines | Hap5 |
| 1449 | 121934 | SPTLR 7201-PRE 26-2-GM-4::IRGC 117356-1 | *indica* | Thailand | Hap5 |
| 1453  wild rice | 121938 | TORO::IRGC 50596-1 | *japonica* | Brazil | Hap5 |
| SRR19177191  SRR19177192 SRR19177197 | MMR01_01#  KHM01_10# 105453 | 'URU WEE' | *O.nivara*  *O.nivara O.nivara* | Myanmar  Cambodia Sri Lanka | Hap7  Hap8 Hap6 |
| SRR19177198 SRR19177213 SRR19177214 SRR19177215 SRR19177216 SRR19177217 SRR19177222 SRR19177223 SRR19177226 SRR19177227 SRR19177255 SRR19177259 SRR19177296  SRR19177299 | 80725 1  SL10_29# SL10_19# SL10_17# SL10_16# SL10_06# NEP06_09# NEP02_34# MMR01_X4# MMR01_X3# 105939 SL11_01# KHM01_X1#  82005 | 'KHAO' PAH' VSR 5 | *O.nivara O.nivara O.nivara O.nivara O.nivara O.nivara O.nivara O.nivara O.nivara O.nivara O.nivara O.nivara O.nivara*  *O.nivara* | Myamar Sri Lanka Sri Lanka Sri Lanka Sri Lanka Sri Lanka Nepal Nepal Myanmar Myanmar Thailand Sri Lanka Cambodia  India | Hap6 Hap8 Hap8 Hap11 Hap8 Hap10 Hap8 Hap10 Hap11 Hap11 Hap8 Hap8 Hap7  Hap6 |
| SRR19177317 | 105318 | 042/87/24 | *O.nivara* | India | Hap8 |
| SRR19177318 SRR19177375 SRR19177383 | 106128  SL11_05# 81940 | MV 89-111  MY 92-W9 | *O.nivara O.nivara O.nivara* | India  Sri Lanka Myanmar | Hap10 Hap11 Hap8 |
| SRR19177385 | 106348 | 'DAUNG SABA' | *O.nivara* | Myanmar | Hap8 |
| SRR19177398 | 105314 | 042/87/20 | *O.nivara* | India | Hap9 |
| SRR19177399 | 105308 | 'VRANELLU' | *O.nivara* | India | Hap11 |
| SRR19177432 | 93188 | NEP 98-061 | *O.nivara* | Nepal | Hap10 |
| SRR19177436 | 80722 | MY 91-3 | *O.nivara* | Myanmar | Hap9 |
| SRR19177439 | 100195 | W 619 | *O.nivara* | Myanmar | Hap6 |
| SRR19177442 | 81858 | JBT 16/D 85 | *O.nivara* | India | Hap10 |
| SRR19177443 | 81857 | JBT 16/D 84 | *O.nivara* | India | Hap8 |
| SRR19177444 | 81856 | JBT 16/D 82 | *O.nivara* | India | Hap11 |
| SRR19177493 | 106345 | 'DAUNG SABA' | *O.nivara* | Myanmar | Hap8 |

| SRR19177494 | 106344 | 'DAUNG SABA' | *O.nivara* | Myanmar | Hap10 |
| --- | --- | --- | --- | --- | --- |
| SRR19177495 | MMR01_23# |  | *O.nivara* | Myanmar | Hap8 |
| SRR19177504 | 81850 | JBT 16/D 54 | *O.nivara* | India | Hap11 |
| SRR19177518 | 100897 | W 106 | *O.nivara* | India | Hap10 |
| SRR19177519 | 89214 | CA 97-014 | *O.nivara* | Cambodia | Hap8 |
| SRR19177106 | NEP07_19# |  | *O.rufipogon* | Nepal | Hap10 |
| SRR19177108 | NEP04_20# |  | *O.rufipogon* | Nepal | Hap6 |
| SRR19177109 | NEP04_04# |  | *O.rufipogon* | Nepal | Hap8 |
| SRR19177112 | MMR05_X19# |  | *O.rufipogon* | Myanmar | Hap8 |
| SRR19177115 | MMR05_X17# |  | *O.rufipogon* | Myanmar | Hap11 |
| SRR19177116 | MMR05_X15# |  | *O.rufipogon* | Myanmar | Hap11 |
| SRR19177117 | KHM02_X9# |  | *O.rufipogon* | Cambodia | Hap8 |
| SRR19177118 | KHM02_X11# |  | *O.rufipogon* | Cambodia | Hap11 |
| SRR19177119 | KHM02_X10# |  | *O.rufipogon* | Cambodia | Hap8 |
| SRR19177124 | 81946 | MY 92-W8 | *O.rufipogon* | Myanmar | Hap7 |
| SRR19177131 | 80658 | VS 76 | *O.rufipogon* | India | Hap10 |
| SRR19177135 | VN02_03# |  | *O.rufipogon* | Laos | Hap8 |
| SRR19177141 | 105942 | 086/88/ST 28 | *O.rufipogon* | Thailand | Hap8 |
| SRR19177142 | 105912 | 'YALAMAN' | *O.rufipogon* | Thailand | Hap9 |
| SRR19177143 | NEP07_14# |  | *O.rufipogon* | Nepal | Hap8 |
| SRR19177149 | SL14_01# |  | *O.rufipogon* | Sri Lanka | Hap8 |
| SRR19177151 | SL02_11# |  | *O.rufipogon* | Sri Lanka | Hap10 |
| SRR19177153 | NEP07_11# |  | *O.rufipogon* | Nepal | Hap8 |
| SRR19177154 | YN_jh_05# |  | *O.rufipogon* | China | Hap8 |
| SRR19177155 | 82000 | 'SEMO' | *O.rufipogon* | Papua New Guinea | Hap8 |
| SRR19177156 | 81995 | 'SEMO' | *O.rufipogon* | Papua New Guinea | Hap9 |
| SRR19177157 | 106505 | PNG 46 | *O.rufipogon* | Papua New Guinea | Hap8 |
| SRR19177158 | 106502 | PNG 24 | *O.rufipogon* | Papua New Guinea | Hap7 |
| SRR19177159 | 106289 | PNG 49 | *O.rufipogon* | Papua New Guinea | Hap8 |
| SRR19177160 | 106286 | 'EKETOP' | *O.rufipogon* | Papua New Guinea | Hap9 |
| SRR19177161 | 106283 | 'EKETOP' | *O.rufipogon* | Papua New Guinea | Hap10 |
| SRR19177164 | 106267 | 'BA' | *O.rufipogon* | Papua New Guinea | Hap8 |
| SRR19177166 | YN_jh_03# |  | *O.rufipogon* | China | Hap8 |
| SRR19177168 | VN13_01# |  | *O.rufipogon* | Laos | Hap6 |
| SRR19177170 | MMR05_11# |  | *O.rufipogon* | Myanmar | Hap6 |
| SRR19177171 | KHM02_01# |  | *O.rufipogon* | Cambodia | Hap7 |
| SRR19177172 | JX_dx_03# |  | *O.rufipogon* | China | Hap8 |
| SRR19177173 | HN_wc_03# |  | *O.rufipogon* | China | Hap8 |
| SRR19177174 | JX_dx_02# |  | *O.rufipogon* | China | Hap7 |
| SRR19177175 | HUN_jy_02# |  | *O.rufipogon* | China | Hap8 |
| SRR19177176 | HUN_jy_01# |  | *O.rufipogon* | China | Hap8 |
| SRR19177177 | HUN_cl_01# |  | *O.rufipogon* | China | Hap8 |
| SRR19177178 | HUN_01# |  | *O.rufipogon* | China | Hap7 |

| SRR19177179 | HN_wc_02# |  | *O.rufipogon* | China | Hap6 |
| --- | --- | --- | --- | --- | --- |
| SRR19177181 | GX_td_01# |  | *O.rufipogon* | China | Hap7 |
| SRR19177182 | GX_hz_02# |  | *O.rufipogon* | China | Hap7 |
| SRR19177185 | GX_bh_03# |  | *O.rufipogon* | China | Hap8 |
| SRR19177186 | GX_bh_02# |  | *O.rufipogon* | China | Hap8 |
| SRR19177187 | GD_qy_01# |  | *O.rufipogon* | China | Hap6 |
| SRR19177193 | 80506 | HK 86 | *O.rufipogon* | India | Hap8 |
| SRR19177200 | GX_qt_01# |  | *O.rufipogon* | China | Hap8 |
| SRR19177229 | 103305 | 315865 | *O.rufipogon* | Philippines | Hap8 |
| SRR19177231 | 93281 | 'OIRI' | *O.rufipogon* | Nepal | Hap10 |
| SRR19177237 | 80742 | 'DAUNG SABA' | *O.rufipogon* | Myanmar | Hap7 |
| SRR19177238 | 106379 | 'DAUNG SABA' | *O.rufipogon* | Myanmar | Hap8 |
| SRR19177242 | 80534 | HK 116 | *O.rufipogon* | India | Hap6 |
| SRR19177243 | 80529 | HK 111 | *O.rufipogon* | India | Hap8 |
| SRR19177247 | 93274 | IJ-W-009 | *O.rufipogon* | Indonesia | Hap6 |
| SRR19177252 | 105303 | OR 51 | *O.rufipogon* | Australia | Hap8 |
| SRR19177253 | 105293 | OR 41 | *O.rufipogon* | Australia | Hap8 |
| SRR19177261 | GX_hz_05# |  | *O.rufipogon* | China | Hap8 |
| SRR19177262 | GX_hz_04# |  | *O.rufipogon* | China | Hap7 |
| SRR19177263 | HN_dz_02# |  | *O.rufipogon* | China | Hap8 |
| SRR19177264 | GX_hz_03# |  | *O.rufipogon* | China | Hap11 |
| SRR19177265 | GX_hz_01# |  | *O.rufipogon* | China | Hap8 |
| SRR19177266 | GX_bs_02# |  | *O.rufipogon* | China | Hap8 |
| SRR19177267 | GX_bh_04# |  | *O.rufipogon* | China | Hap8 |
| SRR19177269 | GD_qy_03# |  | *O.rufipogon* | China | Hap8 |
| SRR19177271 | GD_gz_02# |  | *O.rufipogon* | China | Hap8 |
| SRR19177272 | GD_gz_01# |  | *O.rufipogon* | China | Hap7 |
| SRR19177276 | 89231 | CA 97-057 | *O.rufipogon* | Cambodia | Hap8 |
| SRR19177285 | GX_td_03# |  | *O.rufipogon* | China | Hap8 |
| SRR19177322 | GX_ty_01# |  | *O.rufipogon* | China | Hap8 |
| SRR19177324 | KHM10_12# |  | *O.rufipogon* | Cambodia | Hap8 |
| SRR19177326 | KHM08_05# |  | *O.rufipogon* | Cambodia | Hap8 |
| SRR19177327 | KHM02_11# |  | *O.rufipogon* | Cambodia | Hap9 |
| SRR19177331 | JX_dx_05# |  | *O.rufipogon* | China | Hap8 |
| SRR19177333 | 81980 | MV 89-40 | *O.rufipogon* | India | Hap8 |
| SRR19177334 | FJ_zp_04# |  | *O.rufipogon* | China | Hap10 |
| SRR19177336 | 81881 | JBT 16/D 12 | *O.rufipogon* | India | Hap9 |
| SRR19177337 | 80660 | VS 78 | *O.rufipogon* | India | Hap8 |
| SRR19177338 | 80605 | 'KAPANI' | *O.rufipogon* | India | Hap9 |
| SRR19177342 | 103847 | W 1683 | *O.rufipogon* | India | Hap10 |
| SRR19177345 | 106453 | INDO 89-32 | *O.rufipogon* | Indonesia | Hap8 |
| SRR19177346 | YN_yj_05# |  | *O.rufipogon* | China | Hap8 |
| SRR19177347 | 105958 | 'PADI PADIAN' | *O.rufipogon* | Indonesia | Hap7 |

| SRR19177348 | 105953 | 'PADI PADIAN' | *O.rufipogon* | Indonesia | Hap10 |
| --- | --- | --- | --- | --- | --- |
| SRR19177349 | 105951 | 'PADI PADIAN' | *O.rufipogon* | Indonesia | Hap7 |
| SRR19177350 | 105567 | 'PADI HIJANG' | *O.rufipogon* | Indonesia | Hap10 |
| SRR19177351 | HN_wn_02# |  | *O.rufipogon* | China | Hap8 |
| SRR19177353 | HN_dz_01# |  | *O.rufipogon* | China | Hap8 |
| SRR19177354 | GX_hz_06# |  | *O.rufipogon* | China | Hap8 |
| SRR19177359 | HUN_cl_02# |  | *O.rufipogon* | China | Hap8 |
| SRR19177396 | GD_gz_03# |  | *O.rufipogon* | China | Hap10 |
| SRR19177400 | YN_jh_04# |  | *O.rufipogon* | China | Hap8 |
| SRR19177419 | VN13_03# |  | *O.rufipogon* | Laos | Hap10 |
| SRR19177421 | VN02_14# |  | *O.rufipogon* | Laos | Hap11 |
| SRR19177422 | VN02_06# |  | *O.rufipogon* | Laos | Hap11 |
| SRR19177425 | SL03_15# |  | *O.rufipogon* | Sri Lanka | Hap8 |
| SRR19177437 | KHM14_14# |  | *O.rufipogon* | Cambodia | Hap8 |
| SRR19177448 | FJ_zp_02# |  | *O.rufipogon* | China | Hap8 |
| SRR19177451 | YN_yj_02# |  | *O.rufipogon* | China | Hap11 |
| SRR19177452 | YN_jh_02# |  | *O.rufipogon* | China | Hap11 |
| SRR19177454 | VT05_29# |  | *O.rufipogon* | Vietnam | Hap8 |
| SRR19177455 | VT04_09# |  | *O.rufipogon* | Vietnam | Hap11 |
| SRR19177456 | VT03_07# |  | *O.rufipogon* | Vietnam | Hap8 |
| SRR19177457 | VT02_28# |  | *O.rufipogon* | Vietnam | Hap8 |
| SRR19177458 | VT01_02# |  | *O.rufipogon* | Vietnam | Hap9 |
| SRR19177460 | VN13_31# |  | *O.rufipogon* | Laos | Hap8 |
| SRR19177462 | KHM04_01# |  | *O.rufipogon* | Cambodia | Hap8 |
| SRR19177463 | NEP09_31# |  | *O.rufipogon* | Nepal | Hap8 |
| SRR19177464 | NEP08_26# |  | *O.rufipogon* | Nepal | Hap8 |
| SRR19177465 | NEP08_18# |  | *O.rufipogon* | Nepal | Hap7 |
| SRR19177466 | NEP05_11# |  | *O.rufipogon* | Nepal | Hap10 |
| SRR19177467 | 93277 | NEP-W-034 | *O.rufipogon* | Nepal | Hap8 |
| SRR19177470 | 81990 | 'DAUNG SABA' | *O.rufipogon* | Myanmar | Hap10 |
| SRR19177471 | VN02_04# |  | *O.rufipogon* | Laos | Hap9 |
| SRR19177472 | 106357 | 'DAUNG SABA' | *O.rufipogon* | Myanmar | Hap10 |
| SRR19177473 | MMR32# |  | *O.rufipogon* | Myanmar | Hap9 |
| SRR19177474 | MMR31# |  | *O.rufipogon* | Myanmar | Hap8 |
| SRR19177476 | MMR29# |  | *O.rufipogon* | Myanmar | Hap8 |
| SRR19177477 | MMR27# |  | *O.rufipogon* | Myanmar | Hap8 |
| SRR19177480 | MMR22# |  | *O.rufipogon* | Myanmar | Hap8 |
| SRR19177481 | MMR09_10# |  | *O.rufipogon* | Myanmar | Hap9 |
| SRR19177482 | GX_ty_02# |  | *O.rufipogon* | China | Hap8 |
| SRR19177483 | MMR07_03# |  | *O.rufipogon* | Myanmar | Hap8 |
| SRR19177484 | MMR06_04# |  | *O.rufipogon* | Myanmar | Hap10 |
| SRR19177485 | MMR03_03# |  | *O.rufipogon* | Myanmar | Hap8 |
| SRR19177487 | ML02_06# |  | *O.rufipogon* | Malaysia | Hap8 |

| SRR19177488 | ML01_08# |  | *O.rufipogon* | Malaysia | Hap8 |
| --- | --- | --- | --- | --- | --- |
| SRR19177489 | KHM04_10# |  | *O.rufipogon* | Cambodia | Hap8 |
| SRR19177490 | 106161 | L 89-25 | *O.rufipogon* | Laos | Hap9 |
| SRR19177499  SRR19177521 | YN_jh_06#  GD_gz_04# |  | *O.rufipogon*  *O.rufipogon* | China  China | Hap11  Hap8 |

# ID number and sequences information form Jing et al (2023)
