# Supplementary material for: Natural variation in CTF1 conferring cold tolerance at the flowering stage in rice
Source: Plant Biotechnol J. 2025 Jan 29;23(5):1491–506. doi: 10.1111/pbi.14600 (PMC12018822; doi:10.1111/pbi.14600)
Supplement: Supplementary file 15 — Table S3 The primer sequences used in this study. [file PBI-23-1491-s012.docx]

Table S3 The primer sequences used in this study

Primers Forward (5'-3'） Reverse (5'-3'）

| The primers used for genetic analysis and substitution mapping | | |
| --- | --- | --- |
| ID1F | TTCACGGAGTACAGTTGGTTT | TCACGCCCTGCTAATGTAAT |
| 3I | GAGATTCTGCCAAACCACATT | GAATCTGGCTTTGGCTGTG |
| 3G | TGTGCGTTGGTCTTCGGCT | GCAGAGGGCAATAACAGAATGG |
| 3H | GCAAGCCAGTAGCATTTATGTT | CTGAGGGATGTTCAAAGGAGA |
| 3J | CGTACCTCGATCATAGTTTGTG | ACCGACTCCGTTACGACC |
| ID6-5d | ACATCCCTTTCCCTGCACAC | GATCTGACGGTCACACGACG |
| ID6-5 | TCATGTACGGTAACTTCTCAGCTT | AGCCAGGGGTTTACCAATCC |
| ID6-5e | CGTTCTAGATGCACCCCAA | ATCCGCTACACTGATCACCA |
| ID6-5c | ATATCCCCGGCTGCTATCCT | AAGAGACCACTTGTGACGACG |
| ID6-5m | TACACTCAAAGCACTAAACA | CTAGCTCAACTGCCACCT |
| RM190 | CTTTGTCTATCTCAAGACAC | TTGCAGATGTTCTTCCTGATG |
| ID6-5n | GTAGGTGAAACAGGAGCAA | AACCTTTGAAAATGTGAGATAG |
| ID6-5h | AAGCGCAACTTGACT | AGAACTGGCCCTCC |
| ID6-5f | GAACAAAATGTGGCAAGTCT | GAGGGGATCGAATCTATGAG |
| ID6-5p | GGTAGGTGCACTGGTCGT | AGGGAGGAGGAAGAAGGC |
| ID6-5j | GTGGACGCAGCACAGAGAGA | CTCATCCGACGGACACCATT |
| ID6-7 | TGTGATCAAGTCGTCGTATCG | CCAGTCGTCTGCTAGTGCATG |
| 10I | GCAAGCCCATAGAATGTCAC | CCACGCAAATATGCAACC |
| 10J | TAAGGGTTTGTTTGGTGG | AGAAATGCTGGTCGGTAG |
| 12C | AAATCGAGGATGCTGCTA | GACTATGGAAATGATCGGTAT |
| The primers used for constructing complementary vectors for candidate genes in cold tolerant QTL *qCTF6* | | |
| *LOC_Os06g04200* | CCATGATTACGAATTCtccctttgtcgaggcgttag | TCGGCGCGCCGTCGACccttgttacctccacagccat |
| *LOC_Os06g04210* | CCATGATTACGAATTCgaggattgatttggggattttcc | TCGGCGCGCCGTCGACttcacctacttgcaatgccctta |
| *LOC_Os06g04240* | CCATGATTACGAATTCgctgacctcgctgtgcctatat | TCGGCGCGCCGTCGACtctactccacaaagcgtgctca |

The primers used for constructing *CTF1* over expression vector

*CTF1* -pOX-OV aaaAAGCTTaacctggtcgtgccgtgcgag aaaaACTAGTggatcggaagaacagagcag

The primers used for constructing *CTF1* CRISPR/Cas9 vector

*CTF1* -OsU6aT1 gccgATCATCGCCATGTCCGTGGT aaacACCACGGACATGGCGATGAT

*CTF1* -OsU6bT2 gttgTTCGTCCCGCGGCGGCAAGC aaacGCTTGCCGCCGCGGGACGAA

The primers for constructing sgRNA cassettes to generate A-to-G editing in *CTF1*

AG-U6aT1 gccgACGACGGCGGCGGAAGGCAG aaacCTGCCTTCCGCCGCCGTCGT AG-U6bT2 gttgCAACGCGGGATGGGCTCCTC aaacGAGGAGCCCATCCCGCGTTG

The primers for constructing sgRNA cassettes to generate G-to-A editing in *CTF1*

GA-U6aT1 gccgCCGCCGTCGCCTGTTCCGC aaacGCGGAACAGGCGACGGCGG

GA-U6bT2 gttgCGCTGGATCGAACCGCGCG aaacCGCGCGGTTCGATCCAGCG

The primers for constructing pCTF1::*GUS* vector

*CTF1* -GUS aaaaAAGCTTgaggattgatttggggattttcc aaaaTCTAGAggctcgcacggcacgaccaggttgc

The primers for constructing *CTF1* pGreenII 0800-LUC vectors

*CTF1* -1Kb-LUC TCGACGGTATCGATAAGCTTgaggattgatttggggattttcc GCTCTAGAACTAGTGGATCCggctcgcacggcacgaccaggttgc

The primers for screening the genotype of *CTF1*

*CTF1* -cri AGTCGGATTCCAACATGACGA CCAAAACAAGAGGGGATCGG

The primers for quantitive real time PCR in this study

| *LOC_Os06g04200* -qRT | CCTAGCAAGGACAAGTACATCA | GATCAGTGGGATTTTCCTGTCG |
| --- | --- | --- |
| *LOC_Os06g04210* -qRT | CTACTGCTTCGAGACCATCTC | CACACGCAACCTAAGAAAGAAA |
| *LOC_Os06g04220* -qRT | CCATGGAGAGGTTCTTGACATC | CTTCTTAGCCTCCGATGATGG |
| *LOC_Os06g04230* -qRT | GTTCCCTTCTGACTCTATCTCG | CCACAGCTCTGTACAAATTACG |
| *LOC_Os06g04240* -qRT | CTGGCATTCTCGATCCTGAG | CTAATGCATCACGATGGTCTC |
| *LOC_Os06g04250* -qRT | GAGCCAAGAAAACCACCATTAA | GTACTGCAGCTTGACGAAATC |
| *OsEF1A* | TTTCACTCTTGGTGTGAAGCAGAT | GACTTCCTTCACGATTTCATCGTAA |
