# Supplementary material for: Natural variation in CTF1 conferring cold tolerance at the flowering stage in rice
Source: Plant Biotechnol J. 2025 Jan 29;23(5):1491–506. doi: 10.1111/pbi.14600 (PMC12018822; doi:10.1111/pbi.14600)
Supplement: Supplementary file 16 — Table S4 The 10 mutated CTF1 promoter sequences based on S20‐9‐18 DNA sequences. [file PBI-23-1491-s002.docx]

Table S4 The 10 mutated *CTF1* promoter sequences based on S20-9-18 DNA sequences

Sites Sequences

Var1

GAGGATTGATTTGGGGATTTTCCTACTTGATTCGGGAGAAAATAAAAATGAAAAATGAA AAAGAAGAAGGAACCATCTGCACTACCGCTATTGTCTCTTTAGTCCCGGTTGGTAACAT TAACCGAGACTAAAGATCCCTCTCTTTAGTCCCGGTTGGTGGTACCAACCAGGAATAAA GATGTATCTTTAGTCCCGGTTATTTCAACATTCTTTAATTTTTAATTTTAAATTTCAGTTA CTTCTAAATTGTGTTCCTATATTGCCTTTATACTCTTCTTCCCATATTTTTTTAGATTTTA AATTTTAGTTATTTATAAATTGTATTTTTATACGAATTATAAACTCTACTTTTAATTTTAT TATGTTTATTCCGAATTTTAGTTTGTTTTAAATTCCTATGTGGACTCTATACTCTACTTCT AATATTCCTTATTTTTAATTCCGAATTTATATTATTTCTTAATTGAATTTCTATATGGACT CTATATATACTCTACTTATAATATTCCTTATTTTTAATTCTAAATTTCTATTATTTCTTAA TTATATTTCTATATGGACTCTAGTCTCCTCTTCTAATATTTCTTATTTTTT- AATTCCGAATTTCAACTATTTCTAAATTGTATTTTTATATAGACTCTGTTTTTCTTTTTCT CCGATTAATATGAGAATTTCTAGGCCGCGAGACCGAACGTGGAGGCTCCTTTTTCTATT CTTTTAATAAATTAATAGATAGATATATGTCTAGGTTAATTAATATCA------- TGTGGGAAATGCTAGAATGACTTAGAGGGAGTAGAATTCGTCTCCGAATTACGAGTCGG ATTCCAACATGACGACGAAGCGGCTGTGCCACTGCCGCCGCCTATAAAGCCGGGGGAG GGGGGGGGGG--- GCACGCCACTACGCCACGCGACCTCACCTAACCGCGCGCGCGCCTACCCACCCACGCGA CGCGACGCGCGCGCGGCGTCAGATCGCGGCGGCGAGGCGAGGCGAGTCCGCGCCGCCA GCAACCTGGTCGTGCCGTGCGAGCC

Var2

GAGGATTGATTTGGGGATTTTCCTACTTGATTCGGGAGAAAATAAAAATGAAAAATGAAA AAGAAGAAGGAACCATCTGCACTACCGCTATTGTCTCTTTAGTCCCGGTTGGTAACATTAA CCGAGACTAAAGATCCCTCTCTTTAGTCCCGGTTGGTGGTACCAACCAGGAATAAAGATGT ATCTTTAGTCCCGGTTATTTCAACATTCTTTAATTTTTAATTTTAAATTTCAGTTACTTCTAA ATTGTGTTCCTATATTGCCTTTATACTCTTCTTCCCATATTTTTTTAGATTTTAAATTTTAGTT ATTTATAAATTGTATTTTTATACGAATTATAAACTCTACTTTTAATTTTATTATGTTTATTCC GAATTTTAGTTTGTTTTAAATTCCTATGTGGACTCTATACTCTACTTCTAATATTCCTTATTT TTAATTCCGAATTTATATTATTTCTTAATTGAATTTCTATATGGACTCTATATATACTCTACT TATAATATTCCTTATTTTTAATTCTAAATTTCTATTATTTCTTAATTATATTTCTATATGGACT CTAGTCTCCTCTTCTAATATTTCTTATTTTTT- AATTCCGAATTTCAACTATTTCTAAATTGTATTTTTATATAGACTCTGTTTTTCTTTTTCTCC GATTAATATGAGAATTTCTAGGCCGCGAGACCGAACGTGGAGGCTCCTTTTTCTATTCTTTT AATAAATTAATAGATAGATATATGTCTAGGTTAATTAATATCA------- TGTGGGAAATGCTAGAATGACTTAGAGGGAGTAGAATTCGTCTCCGAATTACGAGTCGGAT TCCAACATGACGACGAAGCGGCTGTGCCACTGCCGCCGCCTATAAAGCCGGGGGAGGGGG GGGGGG---GCACGCCACTACGCCACGCGACCTCAC- CGCCCGCCCACCCACGCGACGCGACGCGCGCGCGGCGTCAGATCGCGGCGGCGAGGCGAG GCGAGTCCGCGCCGCCAGCAACCTGGTCGTGCCGTGCGAGCC

Var3

GAGGATTGATTTGGGGATTTTCCTACTTGATTCGGGAGAAAATAAAAATGAAAAATGAAA AAGAAGAAGGAACCATCTGCACTACCGCTATTGTCTCTTTAGTCCCGGTTGGTAACATTAA CCGAGACTAAAGATCCCTCTCTTTAGTCCCGGTTGGTGGTACCAACCAGGAATAAAGATGT ATCTTTAGTCCCGGTTATTTCAACATTCTTTAATTTTTAATTTTAAATTTCAGTTACTTCTAA ATTGTGTTCCTATATTGCCTTTATACTCTTCTTCCCATATTTTTTTAGATTTTAAATTTTAGTT ATTTATAAATTGTATTTTTATACGAATTATAAACTCTACTTTTAATTTTATTATGTTTATTCC GAATTTTAGTTTGTTTTAAATTCCTATGTGGACTCTATACTCTACTTCTAATATTCCTTATTT TTAATTCCGAATTTATATTATTTCTTAATTGAATTTCTATATGGACTCTATATATACTCTACT TATAATATTCCTTATTTTTAATTCTAAATTTCTATTATTTCTTAATTATATTTCTATATGGACT CTAGTCTCCTCTTCTAATATTTCTTATTTTTT- AATTCCGAATTTCAACTATTTCTAAATTGTATTTTTATATAGACTCTGTTTTTCTTTTTCTCC GATTAATATGAGAATTTCTAGGCCGCGAGACCGAACGTGGAGGCTCCTTTTTCTATTCTTTT AATAAATTAATAGATAGATATATGTCTAGGTTAATTAATATCA------- TGTGGGAAATGCTAGAATGACTTAGAGGGAGTAGAATTCGTCTCCGAATTACGAGTCGGAT TCCAACATGACGACGAAGCGGCTGTGCCGCTGCCGCCGCCTATAAAGCCGGGGGAGGGGG GGGGGG--- GCACGCCACTACGCCACGCGACCTCACCTAACCGCGCGCGCGCCCGCCCACCCACGCGAC GCGACGCGCGCGCGGCGTCAGATCGCGGCGGCGAGGCGAGGCGAGTCCGCGCCGCCAGCA ACCTGGTCGTGCCGTGCGAGCC

Var4

GAGGATTGATTTGGGGATTTTCCTACTTGATTCGGGAGAAAATAAAAATGAAAAATGAA AAAGAAGAAGGAACCATCTGCACTACCGCTATTGTCTCTTTAGTCCCGGTTGGTAACAT TAACCGAGACTAAAGATCCCTCTCTTTAGTCCCGGTTGGTGGTACCAACCAGGAATAAA GATGTATCTTTAGTCCCGGTTATTTCAACATTCTTTAATTTTTAATTTTAAATTTCAGTTA CTTCTAAATTGTGTTCCTATATTGCCTTTATACTCTTCTTCCCATATTTTTTTAGATTTTA AATTTTAGTTATTTATAAATTGTATTTTTATACGAATTATAAACTCTACTTTTAATTTTAT TATGTTTATTCCGAATTTTAGTTTGTTTTAAATTCCTATGTGGACTCTATACTCTACTTCT AATATTCCTTATTTTTAATTCCGAATTTATATTATTTCTTAATTGAATTTCTATATGGACT CTATATATACTCTACTTATAATATTCCTTATTTTTAATTCTAAATTTCTATTATTTCTTAA TTATATTTCTATATGGACTCTAGTCTCCTCTTCTAATATTTCTTATTTTTT- AATTCCGAATTTCAACTATTTCTAAATTGTATTTTTATATAGACTCTGTTTTTCTTTTTCT CCGATTAATATGAGAATTTCTAGGCCGCGAGACCGAACGTGGAGGCTCCTTTTTCTATT CTTTTAATAAATTAATAGATAGATATATGTCTAGGTTAATTAATATCA------- TGTGGGAAATGCTAGAATGACTTAGAGGGAGTAGAATTCATCTCCGAATTACGAGTCGG ATTCCAACATGACGACGAAGCGGCTGTGCCACTGCCGCCGCCTATAAAGCCGGGGGAG GGGGGGGGGG--- GCACGCCACTACGCCACGCGACCTCACCTAACCGCGCGCGCGCCCGCCCACCCACGCGA CGCGACGCGCGCGCGGCGTCAGATCGCGGCGGCGAGGCGAGGCGAGTCCGCGCCGCCA GCAACCTGGTCGTGCCGTGCGAGCC

Var5

Var6

GAGGATTGATTTGGGGATTTTCCTACTTGATTCGGGAGAAAATAAAAATGAAAAATGAAA AAGAAGAAGGAACCATCTGCACTACCGCTATTGTCTCTTTAGTCCCGGTTGGTAACATTAA CCGAGACTAAAGATCCCTCTCTTTAGTCCCGGTTGGTGGTACCAACCAGGAATAAAGATGT ATCTTTAGTCCCGGTTATTTCAACATTCTTTAATTTTTAATTTTAAATTTCAGTTACTTCTAA ATTGTGTTCCTATATTGCCTTTATACTCTTCTTCCCATATTTTTTTAGATTTTAAATTTTAGTT ATTTATAAATTGTATTTTTATACGAATTATAAACTCTACTTTTAATTTTATTATGTTTATTCC GAATTTTAGTTTGTTTTAAATTCCTATGTGGACTCTATACTCTACTTCTAATATTCCTTATTT TTAATTCCGAATTTATATTATTTCTTAATTGAATTTCTATATGGACTCTATATATACTCTACT TATAATATTCCTTATTTTTAATTCTAAATTTCTATTATTTCTTAATTATATTTCTATATGGACT CTAGTCTCCTCTTCTAATATTTCTTATTTTTT- AATTCCGAATTTCAACTATTTCTAAATTGTATTTTTATATAGACTCTGTTTTTCTTTTTCTCC GATTAATATGAGAATTTCTAGGCCGCGAGACCGAACGTGGAGGCTCCTTTTTCTATTCTTTT AATAAATTAATAGATAGATATATGTCTAGGTTAATTAATATCA------- TGTGGGAAATGCTAGAATGACTTGGAGGGAGTAGAATTCGTCTCCGAATTACGAGTCGGAT TCCAACATGACGACGAAGCGGCTGTGCCACTGCCGCCGCCTATAAAGCCGGGGGAGGGGG GGGGGG--- GCACGCCACTACGCCACGCGACCTCACCTAACCGCGCGCGCGCCCGCCCACCCACGCGAC GCGACGCGCGCGCGGCGTCAGATCGCGGCGGCGAGGCGAGGCGAGTCCGCGCCGCCAGCA ACCTGGTCGTGCCGTGCGAGCC

GAGGATTGATTTGGGGATTTTCCTACTTGATTCGGGAGAAAATAAAAATGAAAAATGAAA AAGAAGAAGGAACCATCTGCACTACCGCTATTGTCTCTTTAGTCCCGGTTGGTAACATTAA CCGAGACTAAAGATCCCTCTCTTTAGTCCCGGTTGGTGGTACCAACCAGGAATAAAGATGT ATCTTTAGTCCCGGTTATTTCAACATTCTTTAATTTTTAATTTTAAATTTCAGTTACTTCTAA ATTGTGTTCCTATATTGCCTTTATACTCTTCTTCCCATATTTTTTTAGATTTTAAATTTTAGTT ATTTATAAATTGTATTTTTATACGAATTATAAACTCTACTTTTAATTTTATTATGTTTATTCC GAATTTTAGTTTGTTTTAAATTCCTATGTGGACTCTATACTCTACTTCTAATATTCCTTATTT TTAATTCCGAATTTATATTATTTCTTAATTGAATTTCTATATGGACTCTATATATACTCTACT TATAATATTCCTTATTTTTAATTCTAAATTTCTATTATTTCTTAATTATATTTCTATATGGACT CTAGTCTCCTCTTCTAATATTTCTTATTTTTT- AATTCCGAATTTCAACTATTTCTAAATTGTATTTTTATATAGACTCTGTTTTTCTTTTTCTCC GATTAATATGAGAATTTCTAGGCCGCGAGACCGAACGTGGAGGCTCCTTTTTCTATTCTTTT AATAAATTAATAGATAGATATATGTCTAGGTTAATTAATATCA------- TGTGGGAAATGCTAGAATAACTTAGAGGGAGTAGAATTCGTCTCCGAATTACGAGTCGGAT TCCAACATGACGACGAAGCGGCTGTGCCACTGCCGCCGCCTATAAAGCCGGGGGAGGGGG GGGGGG--- GCACGCCACTACGCCACGCGACCTCACCTAACCGCGCGCGCGCCCGCCCACCCACGCGAC GCGACGCGCGCGCGGCGTCAGATCGCGGCGGCGAGGCGAGGCGAGTCCGCGCCGCCAGCA ACCTGGTCGTGCCGTGCGAGCC

Var7

Var8

GAGGATTGATTTGGGGATTTTCCTACTTGATTCGGGAGAAAATAAAAATGAAAAATGAAA AAGAAGAAGGAACCATCTGCACTACCGCTATTGTCTCTTTAGTCCCGGTTGGTAACATTAA CCGAGACTAAAGATCCCTCTCTTTAGTCCCGGTTGGTGGTACCAACCAGGAATAAAGATGT ATCTTTAGTCCCGGTTATTTCAACATTCTTTAATTTTTAATTTTAAATTTCAGTTACTTCTAA ATTGTGTTCCTATATTGCCTTTATACTCTTCTTCCCATATTTTTTTAGATTTTAAATTTTAGTT ATTTATAAATTGTATTTTTATACGAATTATAAACTCTACTTTTAATTTTATTATGTTTATTCC GAATTTTAGTTTGTTTTAAATTCCTATGTGGACTCTATACTCTACTTCTAATATTCCTTATTT TTAATTCCGAATTTATATTATTTCTTAATTGAATTTCTATATGGACTCTATATATACTCTACT TATAATATTCCTTATTTTTAATTCTAAATTTCTATTATTTCTTAATTATATTTCTATATGGACT CTAGTCTCCTCTTCTAATATTTCTTATTTTTT- AATTCCGAATTTCAACTATTTCTAAATTGTATTTTTATATAGACTCTGTTTTTCTTTTTCTCC GATTAATATGAGAATTTCTAGGCCGCGAGACCGAACGTGGAGGCTCCTTTTTCTATTCTTTT AATAAATTAATAGATAGATATATGTCTAGGTTAATTAATATCA------- TATGGGAAATGCTAGAATGACTTAGAGGGAGTAGAATTCGTCTCCGAATTACGAGTCGGAT TCCAACATGACGACGAAGCGGCTGTGCCACTGCCGCCGCCTATAAAGCCGGGGGAGGGGG GGGGGG--- GCACGCCACTACGCCACGCGACCTCACCTAACCGCGCGCGCGCCCGCCCACCCACGCGAC GCGACGCGCGCGCGGCGTCAGATCGCGGCGGCGAGGCGAGGCGAGTCCGCGCCGCCAGCA ACCTGGTCGTGCCGTGCGAGCC

GAGGATTGATTTGGGGATTTTCCTACTTGATTCGGGAGAAAATAAAAATGAAAAATGAAA AAGAAGAAGGAACCATCTGCACTACCGCTATTGTCTCTTTAGTCCCGGTTGGTAACATTAA CCGAGACTAAAGATCCCTCTCTTTAGTCCCGGTTGGTGGTACCAACCAGGAATAAAGATGT ATCTTTAGTCCCGGTTATTTCAACATTCTTTAATTTTTAATTTTAAATTTCAGTTACTTCTAA ATTGTGTTCCTATATTGCCTTTATACTCTTCTTCCCATATTTTTTTAGATTTTAAATTTTAGTT ATTTATAAATTGTATTTTTATACGAATTATAAACTCTACTTTTAATTTTATTATGTTTATTCC GAATTTTAGTTTGTTTTAAATTCCTATGTGGACTCTATACTCTACTTCTAATATTCCTTATTT TTAATTCCGAATTTATATTATTTCTTAATTGAATTTCTATATGGACTCTATATATACTCTACT TATAATATTCCTTATTTTTAATTCTAAATTTCTATTATTTCTTAATTATATTTCTATATGGACT CTAGTCTCCTCTTCTAATATTTCTTATTTTTT- AATTCCGAATTTCAACTATTTCTAAATTGTATTTTTATATAGACTCTGTTTTTCTTTTTCTCC GATTAATATGAGAATTTCTAGGCCGCGAGACCGAACGTGGAGGCTCCTTTTTCTATTCTTTT AATAAATTAATAGATAGATATATGTCTAGGTTAATTAATATCAATATAAAGTGGGAAATGC TAGAATGACTTAGAGGGAGTAGAATTCGTCTCCGAATTACGAGTCGGATTCCAACATGACG ACGAAGCGGCTGTGCCACTGCCGCCGCCTATAAAGCCGGGGGAGGGGGGGGGGG--- GCACGCCACTACGCCACGCGACCTCACCTAACCGCGCGCGCGCCCGCCCACCCACGCGAC GCGACGCGCGCGCGGCGTCAGATCGCGGCGGCGAGGCGAGGCGAGTCCGCGCCGCCAGCA ACCTGGTCGTGCCGTGCGAGCC

Var9

Var10

GAGGATTGATTTGGGGATTTTCCTACTTGATTCGGGAGAAAATAAAAATGAAAAATGAAA AAGAAGAAGGAACCATCTGCACTACCGCTATTGTCTCTTTAGTCCCGGTTGGTAACATTAA CCGAGACTAAAGATCCCTCTCTTTAGTCCCGGTTGGTGGTACCAACCAGGAATAAAGATGT ATCTTTAGTCCCGGTTATTTCAACATTCTTTAATTTTTAATTTTAAATTTCAGTTACTTCTAA ATTGTGTTCCTATATTGCCTTTATACTCTTCTTCCCATATTTTTTTAGATTTTAAATTTTAGTT ATTTATAAATTGTATTTTTATACGAATTATAAACTCTACTTTTAATTTTATTATGTTTATTCC GAATTTTAGTTTGTTTTAAATTCCTATGTGGACTCTATACTCTACTTCTAATATTCCTTATTT TTAATTCCGAATTTATATTATTTCTTAATTGAATTTCTATATGGACTCTATATATACTCTACT TATAATATTCCTTATTTTTAATTCTAAATTTCTATTATTTCTTAATTATATTTCTATATGGACT CTAGTCTCCTCTTCTAATATTTCTTATTTTTT- AATTCCGAATTTCAACTATTTCTAAATTGTATTTTTATATAGACTCTGTTTTTCTTTTTCTCC GATTAATATGAGAATTTCTAGGCCGCGAGACCGAACGTGGAGGCTCCTTTTTCTATTCTTTT AATAAATTAATAGATAGATATATGTCTAGGTTAATTAACATCA------- TGTGGGAAATGCTAGAATGACTTAGAGGGAGTAGAATTCGTCTCCGAATTACGAGTCGGAT TCCAACATGACGACGAAGCGGCTGTGCCACTGCCGCCGCCTATAAAGCCGGGGGAGGGGG GGGGGG--- GCACGCCACTACGCCACGCGACCTCACCTAACCGCGCGCGCGCCCGCCCACCCACGCGAC GCGACGCGCGCGCGGCGTCAGATCGCGGCGGCGAGGCGAGGCGAGTCCGCGCCGCCAGCA ACCTGGTCGTGCCGTGCGAGCC

GAGGATTGATTTGGGGATTTTCCTACTTGATTCGGGAGAAAATAAAAATGAAAAATGAAA AAGAAGAAGGAACCATCTGCACTACCGCTATTGTCTCTTTAGTCCCGGTTGGTAACATTAA CCGAGACTAAAGATCCCTCTCTTTAGTCCCGGTTGGTGGTACCAACCAGGAATAAAGATGT ATCTTTAGTCCCGGTTATTTCAACATTCTTTAATTTTTAATTTTAAATTTCAGTTACTTCTAA ATTGTGTTCCTATATTGCCTTTATACTCTTCTTCCCATATTTTTTTAGATTTTAAATTTTAGTT ATTTATAAATTGTATTTTTATACGAATTATAAACTCTACTTTTAATTTTATTATGTTTATTCC GAATTTTAGTTTGTTTTAAATTCCTATGTGGACTCTATACTCTACTTCTAATATTCCTTATTT TTAATTCCGAATTTATATTATTTCTTAATTGAATTTCTATATGGACTCTATATATACTCTACT TATAATATTCCTTATTTTTAATTCTAAATTTCTATTATTTCTTAATTATATTTCTATATGGACT CTAGTCTCCTCTTCTAATATTTCTTATTTTTT- AATTCCGAATTTCAACTATTTCTAAATTGTATTTTTATATAGACTCTGTTTTTCTTTTTCTCC GATTAATATGAGAATTTCTAGGCCGCGAGACCGAACGTGGAGGCTCCTTTTTCTATTCTTTT AATAAATTAATAGATAGATATATGTCTAGATTAATTAATATCA------- TGTGGGAAATGCTAGAATGACTTAGAGGGAGTAGAATTCGTCTCCGAATTACGAGTCGGAT TCCAACATGACGACGAAGCGGCTGTGCCACTGCCGCCGCCTATAAAGCCGGGGGAGGGGG GGGGGG--- GCACGCCACTACGCCACGCGACCTCACCTAACCGCGCGCGCGCCCGCCCACCCACGCGAC GCGACGCGCGCGCGGCGTCAGATCGCGGCGGCGAGGCGAGGCGAGTCCGCGCCGCCAGCA ACCTGGTCGTGCCGTGCGAGCC
