# Supplementary material for: Natural variation in CTF1 conferring cold tolerance at the flowering stage in rice
Source: Plant Biotechnol J. 2025 Jan 29;23(5):1491–506. doi: 10.1111/pbi.14600 (PMC12018822; doi:10.1111/pbi.14600)
Supplement: Supplementary file 17 — Table S5 Average nucleotide diversity of CTF1 and 20 kb flanking region. [file PBI-23-1491-s010.docx]

Table S5. Average nucleotide diversity of *CTF1* and 20 kb flanking region.

| Loci | *aus* | *indica* | Subpopulati  *japonica* | ons  *O.nivara* | *O.rufipogon* |
| --- | --- | --- | --- | --- | --- |
| Upstream 20kb | 0.002 | 0.0023 | 0.00034 | 0.00502 | 0.0017 |
| *CTF1* | 0.002 | 0.0041 | 0.00035 | 0.01083 | 0.001 |
| Downstream 20kb | 0.0017 | 0.0022 | 0.00037 | 0.00521 | 0.0036 |
