# Supplementary material for: Natural variation in CTF1 conferring cold tolerance at the flowering stage in rice
Source: Plant Biotechnol J. 2025 Jan 29;23(5):1491–506. doi: 10.1111/pbi.14600 (PMC12018822; doi:10.1111/pbi.14600)
Supplement: Supplementary file 18 — Table S6 Neutrality tests of CTF1. [file PBI-23-1491-s018.docx]

Table S6.Neutrality tests of *CTF1*

| Taxon | N | Tajima's *D* |
| --- | --- | --- |
| *japonica* | 124 | -2.02* |
| *indica* | 136 | -0.52 |
| *aus* | 47 | -0.51 |

N, total number of samples.*, *P* < 0.05
